# Emergent Optical Resonances in Atomically Phase-Patterned Semiconducting Monolayers of WS_2_

**DOI:** 10.1021/acsphotonics.4c00983

**Published:** 2024-08-16

**Authors:** John M. Woods, Saroj B. Chand, Enrique Mejia, Ashok Adhikari, Takashi Taniguchi, Kenji Watanabe, Johannes Flick, Gabriele Grosso

**Affiliations:** † Photonics Initiative, Advanced Science Research Center, City University of New York, New York, New York 10031, United States; ‡ International Center for Materials Nanoarchitectonics, National Institute for Materials Science, 1-1 Namiki, Tsukuba 305-0044, Japan; § Research Center for Functional Materials, National Institute for Materials Science, 1-1 Namiki, Tsukuba 305-0044, Japan; ∥ Center for Computational Quantum Physics, Flatiron Institute, New York, New York 10010, United States; ⊥ Department of Physics, City College of New York, New York, New York 10031, United States; # Physics Program, Graduate Center, City University of New York, New York New York 10016, United States

**Keywords:** light–matter interactions, 2D materials, atomically sharp interfaces, transition metal dichalcogenides, WS_2_, excitons, phase transitions

## Abstract

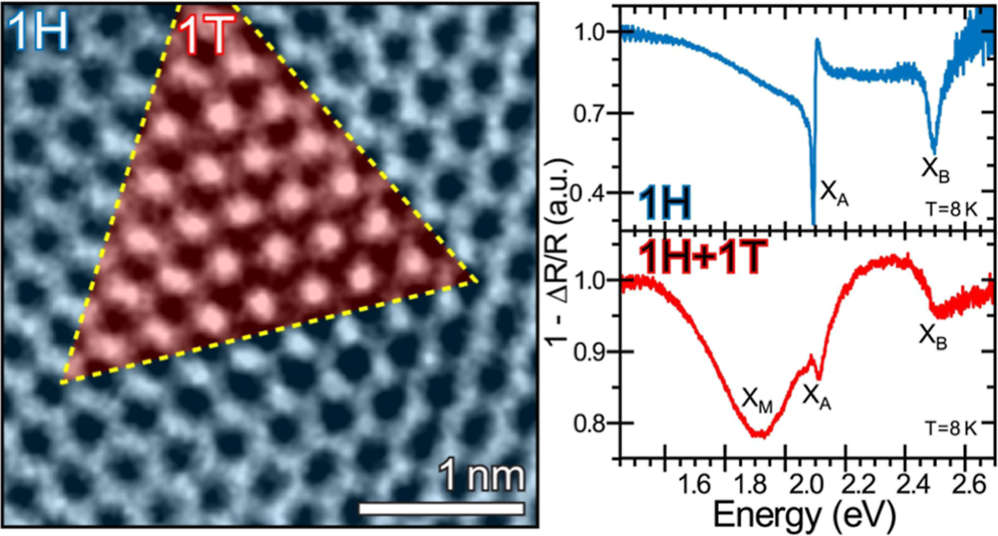

Atomic-scale control of light–matter interactions represents the ultimate frontier for many applications in photonics and quantum technology. Two-dimensional semiconductors, including transition-metal dichalcogenides, are a promising platform to achieve such control due to the combination of an atomically thin geometry and convenient photophysical properties. Here, we demonstrate that a variety of durable polymorphic structures can be combined to generate additional optical resonances beyond the standard excitons. We theoretically predict and experimentally show that atomic-sized patches of the 1T phase within the 1H matrix form unique electronic bands that lead to the emergence of robust optical resonances with strong absorption, circularly polarized emission, and long radiative lifetimes. The atomic manipulation of two-dimensional semiconductors opens unexplored scenarios for light harvesting devices and exciton-based photonics.

## Introduction

Light–matter interactions are the underlying mechanisms of a wide range of scientific disciplines, and they have unlocked a series of key technological advancements. Understanding and controlling such interactions at the atomic scale would open a window on unexplored phenomena, allowing for unprecedented levels of device scalability. However, engineering atomic-scale systems is challenging and requires suitable platforms. Transition-metal dichalcogenides (TMDs) have proven to be advantageous materials for a variety of applications in photonics, electronics, and chemistry due to their malleability resulting from the atomically thin geometry combined with convenient electronic band structures.^1,2^ The latter leads to robust excitonic resonances imbued with spin-valley locking, large oscillator strength, and a large binding energy. In the semiconducting (1H) phase, monolayers of TMDs have a bandgap in the visible range of the spectrum that gives rise to several bright^3,4^ and dark exciton states.^5−7^ The splitting of the valence band resulting from the large spin–orbit coupling (SOC) generates two exciton resonances at the K symmetry point of the Brillouin zone. These are the well-known A and B excitons. The energy difference between these two excitons depends on the type of TMD, with the A excitons being the excitonic ground state of the system. Another peak, corresponding to the C exciton, appears in the absorption spectrum of TMDs and emerges from a band nesting effect.^8−10^

Besides optical properties, TMDs have attracted growing interest for their mechanical and chemical properties, including polymorphism. For example, transition-metal disulfides, such as MoS_2_ and WS_2_, primarily exist in the thermodynamically favored semiconducting 1H phase, but can be forced to the 1T phase with metallic properties through bottom-up growth^11,12^ or top-down treatment^13,14^ pathways. In the 1H phase, both S atoms are located on top of each other (when viewed along the *c*-axis), resulting in a trigonal prismatic symmetry about the metal atom, whereas in the 1T phase, the two S atoms are displaced into an octahedral symmetry, as shown in Supporting Information Figure S1. Transitions from the 1H to the 1T phase (1H → 1T) can be triggered by weak external perturbations, such as chemical, thermal, and irradiation treatments, and the resulting phase boundaries have found several applications in electronics and catalysis.^15−17^ However, the optical properties and possible photonic applications of phase boundaries and mixed-phase and atomic-sized phase structures in two-dimensional materials have not been exhaustively explored yet.

Here, we demonstrate that additional optical transitions can occur across the 1H/1T phase boundaries, in which the metallic 1T phase provides electrons that are optically excited toward an unoccupied conduction band of the semiconducting 1H phase. By using monolayers of WS_2_ as a blank canvas, we sculpt mixed-phased structures with atomic-size patterns and show that optical resonances appear in parts of the spectrum that are naturally inaccessible in pristine 1H materials. We unveil the emergence of an additional peak in both differential reflectance and photoluminescence (PL) emission spectra, which we refer to as the M band because it stems from the mixed phase structure. Ab initio calculations based on density functional theory (DFT) pinpoint the orbital origin of the M band, and microphotoluminescence experiments reveal promising optical properties, including room temperature stability, large oscillator strength, long lifetime, and circular polarization. The demonstration of the creation of resonances through atomic manipulations of TMDs paves the way toward the engineering of their full optical spectrum and the realization of atomic-size devices.

## Atomic-Size Phase Patterns in WS_2_

**Figure 1:**
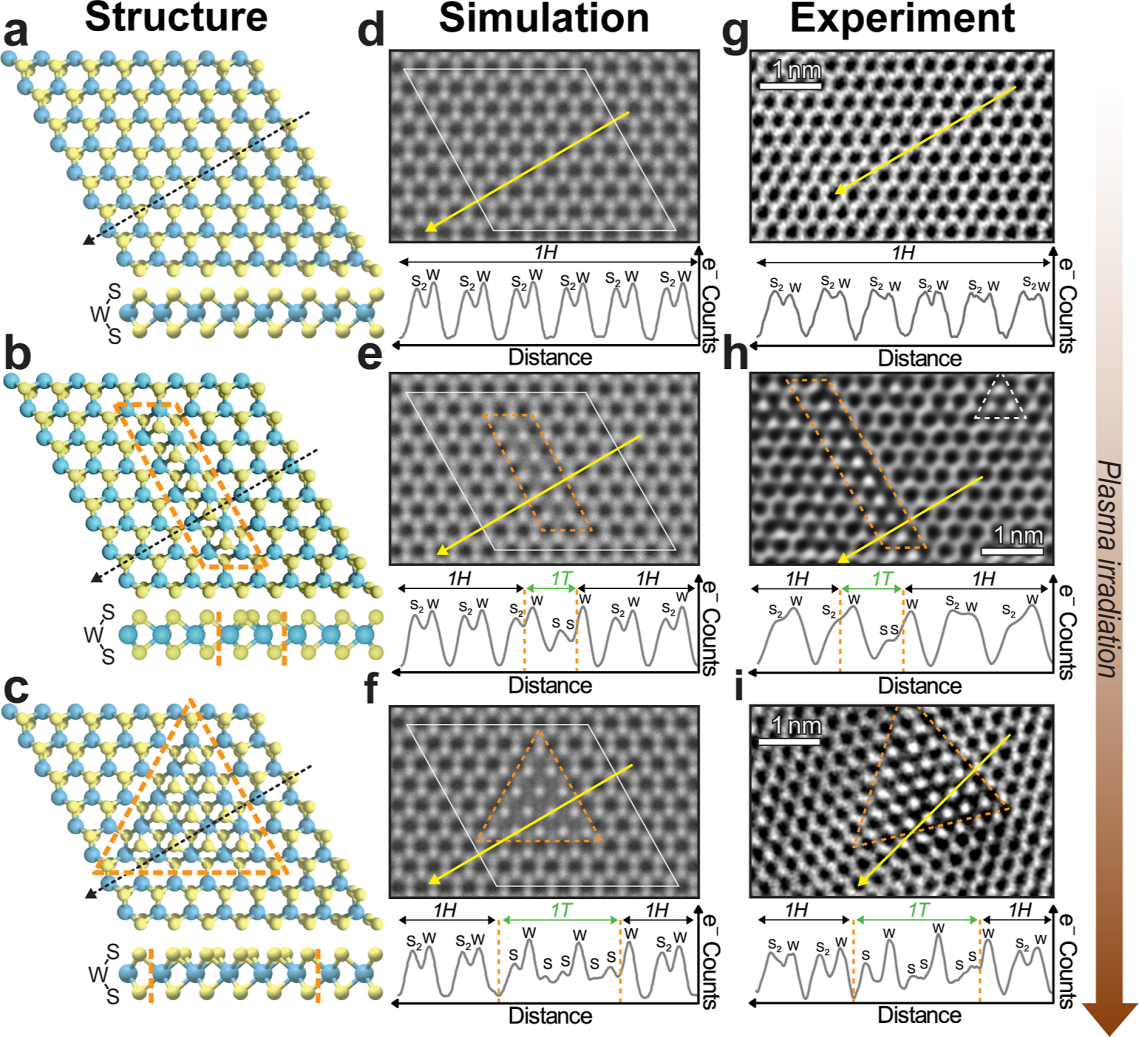
Mixed-phase atomic structures of WS_2_. Top and side views of the atomic structure of (a) 1H pristine, (b) linear 1T/1H (1T-L1), and (c) triangular 1T/1H (1T-Tr4) mixed phase structures of WS_2_ monolayers. Orange, dashed lines indicate the extent of 1T grain, and black dashed lines indicate equivalent path to intensity line profiles in (d–i). Simulated TEM images (d–f) of the structures in (a–c), indicated by a thin white line, surrounded by extra pristine 1H-WS_2_. The bottom trace of each panel indicates the intensity line profile along the path highlighted by the yellow arrow. There is a noticeable difference between S_2_ stacks in 1H-WS_2_ and isolated sulfurs in 1T-WS_2_. Experimental TEM images of pristine WS_2_ (g) and WS_2_ flakes treated with argon plasma (see Methods) for 5 s (h) and 10 s (i). The bottom trace of each panel is taken along the path indicated by the yellow arrow, showing good agreement between the experiment and simulated TEM images. WS_2_ treated for 5 s primarily contains linear 1T grains (see Supporting Information Figure S2 for lower magnification TEM images), but we can also see small triangular grains indicated in (h) by a white dashed line. As the plasma treatment time is increased further, the phase mosaic is dominated by larger triangular and polygonal grains, as demonstrated in (i).

In TMDs, the 1H → 1T phase transition occurs through the collective displacements of atoms and involves stable intermediate structures that eventually merge together forming the final 1T atomic lattice geometry.^18^ When driven by external stimuli, this phase transition can be interrupted midway, generating mixed-phase states in which atomic-size 1T grains are assembled in the 1H matrix with peculiar phase boundaries. The size of the grains can be controlled by tuning the duration and strength of the external stimulus used to trigger the phase transition. We create phase mixture states by irradiating monolayers of WS_2_ with argon plasma. This technique has previously been used to achieve a full metallic phase and to macroscopically pattern different phases in TMDs.^13^ In our experiments, the plasma power and the irradiation time are tuned such that the treatment does not fully convert the pristine 1H monolayers (Methods) but generates instead 1T structures of different shapes and sizes (from less than half a nanometer wide to a few nanometers across) embedded within the broader 1H phase, as shown in Figures 1 and S2. Figure 1a–c illustrates the atomic models of the intermediate steps of the phase transition achieved by plasma irradiation, while Figure 1d–f shows simulations of how the same structures would look in atomically resolved electron images. Figure 1g–i shows experimental images of the atomic lattice of a pristine monolayer (Figure 1g) and plasma-treated monolayers with an irradiation time of 5 s (Figure 1h) and 10 s (Figure 1i). Atomically resolved images of the lattice are obtained by high-resolution transmission electron microscopy (TEM) taken with focusing conditions for bright atom contrast that allow us to clearly resolve W and S atoms, as well as S vacancies and different phases. Line profiles (taken along the direction indicated by yellow arrows in Figure 1d–i) allow us to identify the 1H and 1T phase regions.^14,19,20^

The effect of the plasma is 2-fold: it creates S vacancies and, at the same time, provides the shear force and energy responsible for the shift of the S atoms out of their thermodynamically favored 1H positions toward the 1T phase.^14,18,21^ Analysis of experimental TEM images of sample irradiated for 5 s reveals the presence of intermediate, linear 1T structures with a lateral dimension of 1 lattice vector near a line of S vacancies. Similar structures were identified as precursors of the 1T phase in the pioneering studies on electron-driven phase transitions in TMDs.^18^ Simulated TEM images of a linear 1T structure within 1H-WS_2_ (Figure 1e) are consistent in appearance and show a line profile similar to that of the experimentally observed 1T grain in Figure 1h. To differentiate from other structures, we will refer to these 1T grains with linear morphologies and varying length as 1T-L1. A detailed discussion on the characterization of these structures is in Supporting Information Figure S3. Less frequently, we observe in the samples treated for 5 s the presence of small 1T triangular grains. Longer treatments give rise to larger triangular or polygonal grains of the 1T phase surrounded by the 1H phase (Figure 1i). We refer to the triangular grains as 1T-Tr*N*, where *N* indicates the lateral size in the lattice vectors of the 1T triangle. Moreover, electron diffraction patterns (Supporting Information Figure S4) taken from a large area (∼18,000 nm^2^) of the plasma-treated sample maintain the 6-fold symmetry of pristine 1H-WS_2_, indicating both the absence of high-angle grain boundaries due to a perfect register of the induced 1T phases to the remaining regions of 1H phase as well as further confirming the resulting phase is the 1T phase, and not the 1T′ phase (extensively studied in telluride TMDs), which would instead have a rectangular diffraction pattern.^22^ The partial phase transition with plasma irradiation and copresence of 1H and 1T phases in the treated samples are further confirmed by Raman and X-ray photoelectron (XPS) spectroscopy (Supporting Information Note 1).

## Band Structure and Optical Properties of Mixed-Phase Patterns

**Figure 2:**
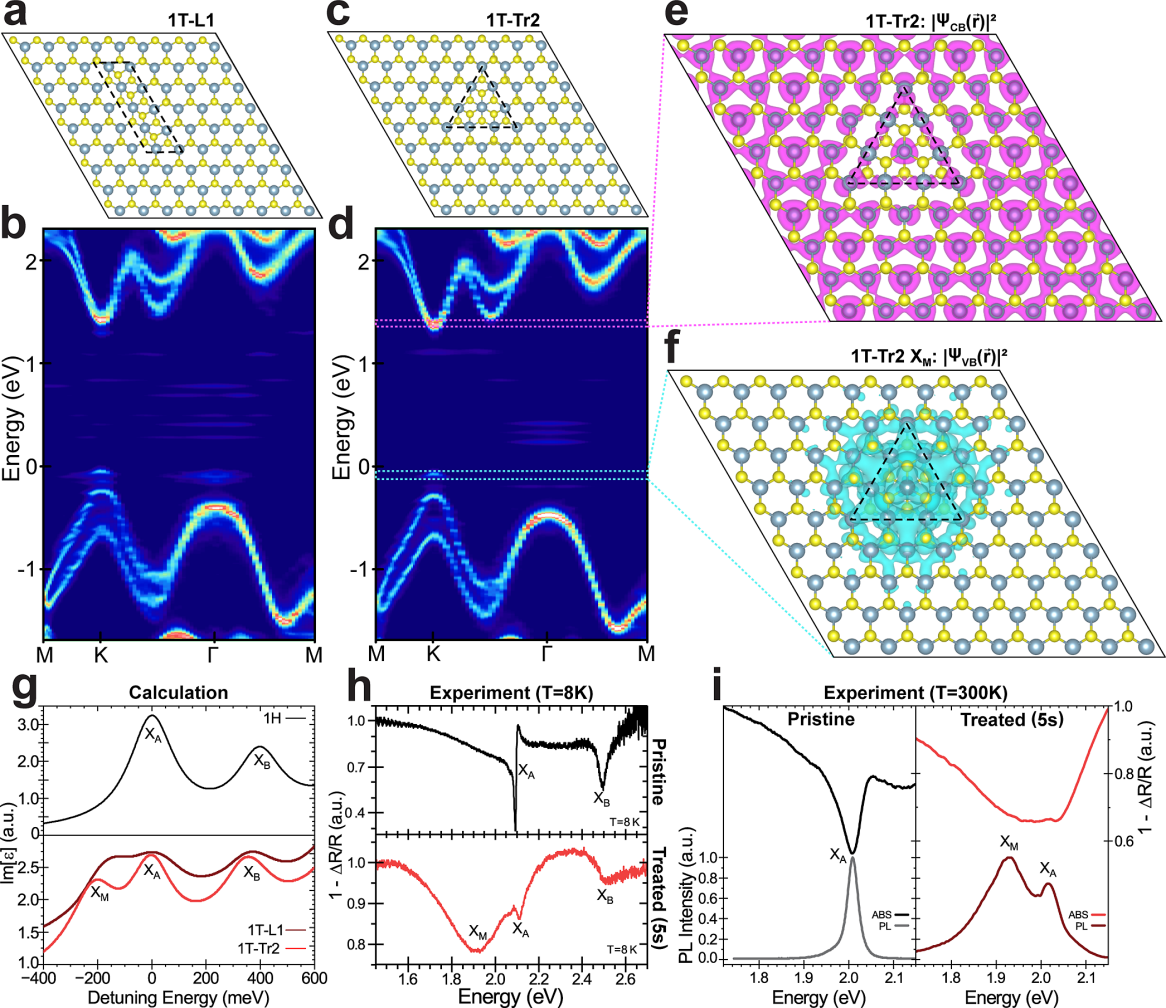
Electronic and optical properties of mixed-phase atomic structures. 9 × 9 × 1 relaxed supercell with linear 1T-L1 (a) and triangular 1T-Tr2 (c) structures of 1T phase within a WS_2_ monolayer in the 1H phase. (b,d) Corresponding band structure calculated with DFT (see Methods) for these mixed-phase supercells. The color scale in (b,d) represents the spectral weight of the band structure at a given momentum-energy coordinate. Real space extent of the pseudowave function of the conduction (e) and valence (f) states involved in the M transition for the 1T-Tr2 structure. The orbitals of the valence band are localized around the 1T phase, while the ones of the conduction band are delocalized across the 1H phase domain. (g) Calculated imaginary part of the dielectric function for 1H pristine (black curve), 1T-Tr2 (red curve), and 1T-L1 (maroon curve). The M resonance (*X*_M_) emerges below the A and B excitons. An arbitrary Gaussian broadening of ∼140 meV is applied to all dielectric calculations from processing DFT results with the Sumo package. (h) Differential reflectivity measurements at *T* = 8 K of pristine (black curve) and mixed-phase (red curve) samples treated with plasma for 5 s, indicating a good agreement with theoretical calculations of (g). (i) Comparison of the absorption (top plots) and emission (bottom plots) spectra of pristine (black curves) and 5 s-treated mixed-phase (red curves) samples at room temperature. Again, the M band appears below the A exciton in both the absorption and emission spectra.

The atomic-size lateral confinement of the 1T phase within the 1H areas dramatically alters the electronic and optical properties of WS_2_ due to the formation of additional electronic states within the standard optical band gap of the 1H phase. The results of ab initio calculations using DFT on 9 × 9 × 1 supercells containing 1T/1H mixed phase structures similar to the ones observed in the TEM images are shown in Figure 2. First, we look at the band structure (obtained using band-folding; see Methods) of the 1T-L1 (Figure 2a,b) and 1T-Tr2 (Figure 2c,d) within a 1H matrix. The presence of 1T-L1 or 1T-Tr2 within the 1H phase introduces occupied electronic states in WS_2_ just above the 1H valence band edge. These states together with the 1H K-valley conduction band host an optical transition with a large dipole moment at an energy below that of the A exciton. The corresponding orbitals (or Kohn–Sham wave functions) in real space for this pair of states in the 1T-Tr2 supercell are shown in Figure 2e,f. Clearly, the occupied state of the transition (Figure 2f) is localized around the triangular region and is therefore primarily attributed to the 1T phase. The electrons provided by the metallic phase can be photoexcited toward unoccupied states (Figure 2e) whose orbitals are delocalized in the 1H semiconducting phase. The shape and distribution of this orbital, in addition to its location in the electronic band structure, allow us to identify this state as the conduction band edge at the K valley of the 1H phase (Supporting Information Figure S8). Calculations performed on 1T-L1 return similar results regarding the nature of the orbitals (Supporting Information Figure S9). Figure 2g compares the calculated imaginary part of the dielectric function for a full 1H supercell, the 1T-Tr2, and the 1T-L1 structures indexed to the energy of the A exciton transition. The higher energy peak is the B exciton, emerging from the splitting of the band edges at the K-valley due to SOC. In the 1T-Tr2 and 1T-L1 structures (red and maroon curves, respectively), another resonance, with a strength comparable to the A and B excitons (*X*_A_ and *X*_B_), appears around 200 meV below the A exciton. This additional resonance is related to the occupied midgap states emerging from the 1H/1T mixed phase structure (Figure 2f) as previously discussed, and we refer to it as the M band (*X*_M_). For the sake of stability and for a realistic comparison with the experimental observations and the known role of vacancy formation to the 1H → 1T phase transformation,^14^ we use an atomic 1T-L1 structure that contains a line of vacancies at one of the two boundaries between the 1H and 1T phase. The relaxation of 1T-L1 and 1T-Tr2 structures without vacancies resulted in a return to a fully pristine 1H supercell. Remarkably, we note that these new bands that give rise to the M band are unique to the mixed-phase structures and cannot be ascribed to the constituent 1H and 1T phases, or the lines of vacancies, necessary to form a stable structure (Supporting Information Figure S10). In the calculated band structures, other localized, but unoccupied, states appear within the gap, and they can be associated with vacancies and the small 1T grains.

We perform optical experiments on samples containing mixed-phase structures after transferring thin layers (∼20 nm) of hexagonal boron nitride (hBN) onto the treated WS_2_ monolayers. Top encapsulation results in the narrowing of the emission lines^23^ and, at the same time, suggests that the mixed-phase samples can be integrated into more complex heterostructures. Differential reflectivity spectra in Figure 2h measured at cryogenic temperature (*T* = 8 K) for pristine and 5 s-treated samples confirm the generation of an additional optical resonance. While pristine WS_2_ shows the typical reflectivity spectra of the 1H phase in this energy range with the A exciton and B exciton peaks, the treated samples hosting mixed-phase atomic structures reveal the appearance of a pronounced peak associated with the M band at ∼200 meV below the A exciton. The absorption of the M band dominates the one of the A exciton in treated samples. We attribute the decrease of absorption of the A and B excitons in treated samples with respect to the pristine case to the reduction of the coverage of the 1H phase as a consequence of the formation of 1T patches as well as an increased defect density in the atomic lattice upon plasma irradiation.^24,25^ The good agreement between the theoretical and experimental values of the energy of the M band relative to the A exciton indicates that this peak is associated with the mixed-phase atomic structures. The broad line width of the M absorption peak can be attributed to the presence in the 5 s-treated samples of multiple mixed-phase structures with different shapes and interfaces. In fact, we observe a predominant presence of 1T-L1 structures and a minority of small triangles (Figures 1h and S2). As discussed below, different structures give rise to M transitions with varying energy. The emission and differential reflectivity spectra of the pristine and 5 s-treated samples at room temperature are shown in Figure 2i. At room temperature, the intensity of the differential reflectivity of the A and M peaks is comparable at around 0.6, while the area of the peak associated with the M band is larger than the A exciton, suggesting a stronger oscillator strength.^26^ Furthermore, the emission intensity of the M band dominates the one of the A exciton in the PL spectrum, indicating that the former represents the preferred relaxation pathways in mixed-phase samples.

**Figure 3:**
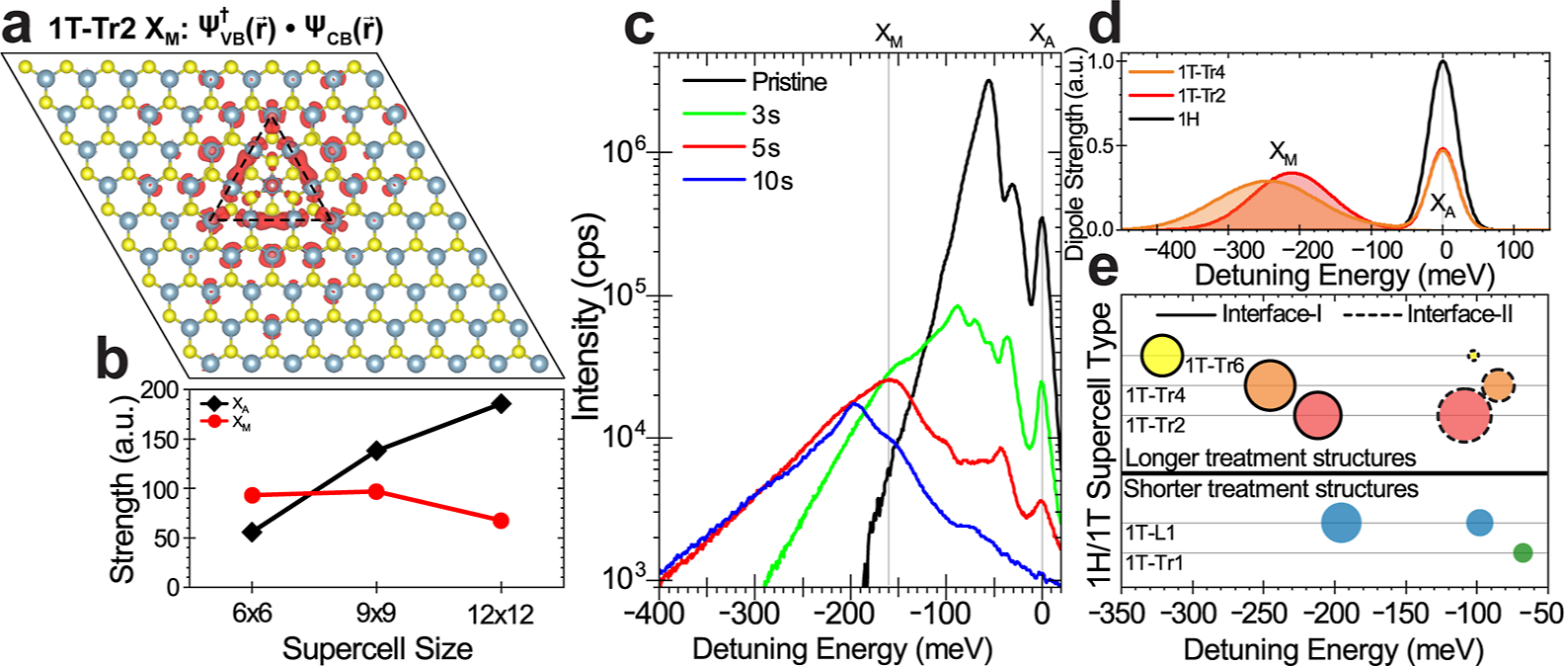
Atomic origin of the M band in mixed-phase atomic structures of different sizes. (a) Calculated transition density of the M band in a 1T-Tr2 supercell, which is concentrated along the interface between the triangular 1T grain and the surrounding 1H phase. (b) Calculated dipole strength of *X*_A_ and *X*_M_ resonances in 1T-Tr2 supercells of increasing size. The strength of *X*_A_ grows as larger supercells have more 1H-WS_2_. In contrast, the strength of *X*_M_ remains almost constant throughout, a further indication of the interfacial nature of the M band. (c) Emission spectra at *T* = 8 K taken with the same excitation laser power of pristine and mixed-phase samples treated for 3, 5, and 10 s. Spectra are indexed at the energy of the A exciton. (d) Plot of the calculated dipole strength as a function of detuning energy for *X*_A_ and *X*_M_ in 1H (black), 1T-Tr2 (red), and 1T-Tr4 (orange) supercells. For legibility, constituent transition dipoles are represented by a Gaussian distribution. (e) Summary of the energy and strength of the transition dipoles extracted from DFT calculations on 9 × 9 × 1 supercells containing different atomic-sized mixed-phase structures. The size of bubbles indicates the strength of the *X*_M_ resonance, represented in (d) as the area under the *X*_M_ peak. Dashed circles highlight the results of mixed-phase structures with type II interfaces. The dipole transition energy is plotted as a function of the detuning energy of the A exciton. An overall red shift of the M band occurs as a function of the size of the 1T grain, in agreement with the experimental data.

Having confirmed the emergence of an additional optical resonance in mixed-phase structures, a discussion of how the optical transition occurs across the two phases is in order. Although the occupied and unoccupied states giving rise to the M band show mostly 1T and 1H characters, respectively, a weak hybridization of the electronic bands occurs at the 1H/1T interface. Moreover, the spatial extent of the transition density, ψ_VB_^†^ (*r⃗*)·ψ_CB_ (*r⃗*), shown in Figure 3a, indicates that the overlap is greatest at the interface where the optical transition is most likely to take place. In contrast, the transition density for the *X*_A_ transition (Supporting Information Figure S11) is delocalized across the 1H regions of the supercell. The role of the interfaces is further confirmed by the calculation of the strength of the transition dipole moments as a function of supercell size. Figure 3b shows that while the strength of the A exciton in a 1T-Tr2 mixed-phase structure increases with increasing supercell size, the strength of the M band is almost constant.

We then characterize the optical properties of samples treated for different times, therefore hosting different atomic mixed-phase structures. Figure 3c shows the emission spectrum at *T* = 8 K of pristine, 3 s-, 5 s-, and 10 s-treated WS_2_ monolayers taken at equivalent excitation power. In pristine WS_2_, we identify the usual emission from A exciton complexes that appear in n-doped samples that include in descending energy the neutral exciton, triplet and singlet trions, charged biexcitons, and complexes related to dark excitons.^5,6,27^ An extensive discussion on the attribution of these peaks is in Supporting Information Note 3. We observe that the emission intensity of all of the A exciton complexes reduces as a function of the treatment time due to a progressive reduction of the 1H phase and the formation of the 1T grains. At 5 s, emission from the M band, peaking around 165 meV below the neutral A exciton, becomes the dominant feature in the spectra, indicating a large density of 1T-L1 and 1T-Tr2 structures in the samples. The emission spectrum of the 10 s-treated sample shows the strongest emission at an even lower energy. As observed in the TEM images in Figure 1, the mixed phase structure switches from linear and small triangular 1T grains to larger triangular 1T grains when the treatment time is increased to 10 s. To understand the role of the 1T grain size, we perform a theoretical analysis on supercells containing triangle 1T grains of increasing size (see Supporting Information Figure S12 for the atomic structures). When we extract the energy and transition dipole strength of the M transitions in these structures (Figure 3d), we observe an overall red shift and strength reduction when increasing the size of the 1T triangles. Figure 3e summarizes the evolution of the M band for these larger triangle cells as compared to the 1T-L1 and 1T-Tr2 mixed phased structures. These results are in good agreement with the experiments and indicate that the energy shift seen for the samples with the longest treatment time is due to the change in the size of the 1T grains. Moreover, Figure 3e shows that each mixed-phase structure generates more than one M resonance with different energies and strengths, depending on the phase interfaces involved in the atomic structure. In fact, while 1T triangular structures are separated from the 1H matrix by only one kind of phase interface, 1T lines contain two inequivalent phase interfaces on either side of the 1T grain with different arrangements of S atoms around border W atoms (see Supporting Information Figure S13). The presence of distinct boundaries on either side of 1T-L1 explains the observation in Figure 3e of two M resonances with the orbitals illustrated in Supporting Information Figure S9. Except for 1T-Tr1, the phase boundaries of 1T-Tr*N* change with the relative orientation of the triangle within the 1H matrix. The 1T-Tr2 structure in Figure 2 has Interface-I (Supporting Information Figure S13a) edges, whereas Figure S14 in the Supporting Information reports the results of the calculations for a 1T-Tr2 structure with the opposite orientation, Interface-II (Figure S13b), with respect to the 1H phase. For this structure, the M band appears at a higher energy compared with the Interface-I 1T-Tr2 structure shown in Figure 2. In Figure 3e, we differentiate the results of theoretical calculations for 1T-Tr*N* for both orientations, with a dashed line border representing the Interface-II structures. We note that the energy dependence on the orientation and size of the 1T phase relative to the 1H matrix explains the broad line width of the M resonance experimentally observed in our treated samples that contain diverse atomic-size structures.

## Optical Characterization of the M Band

**Figure 4:**
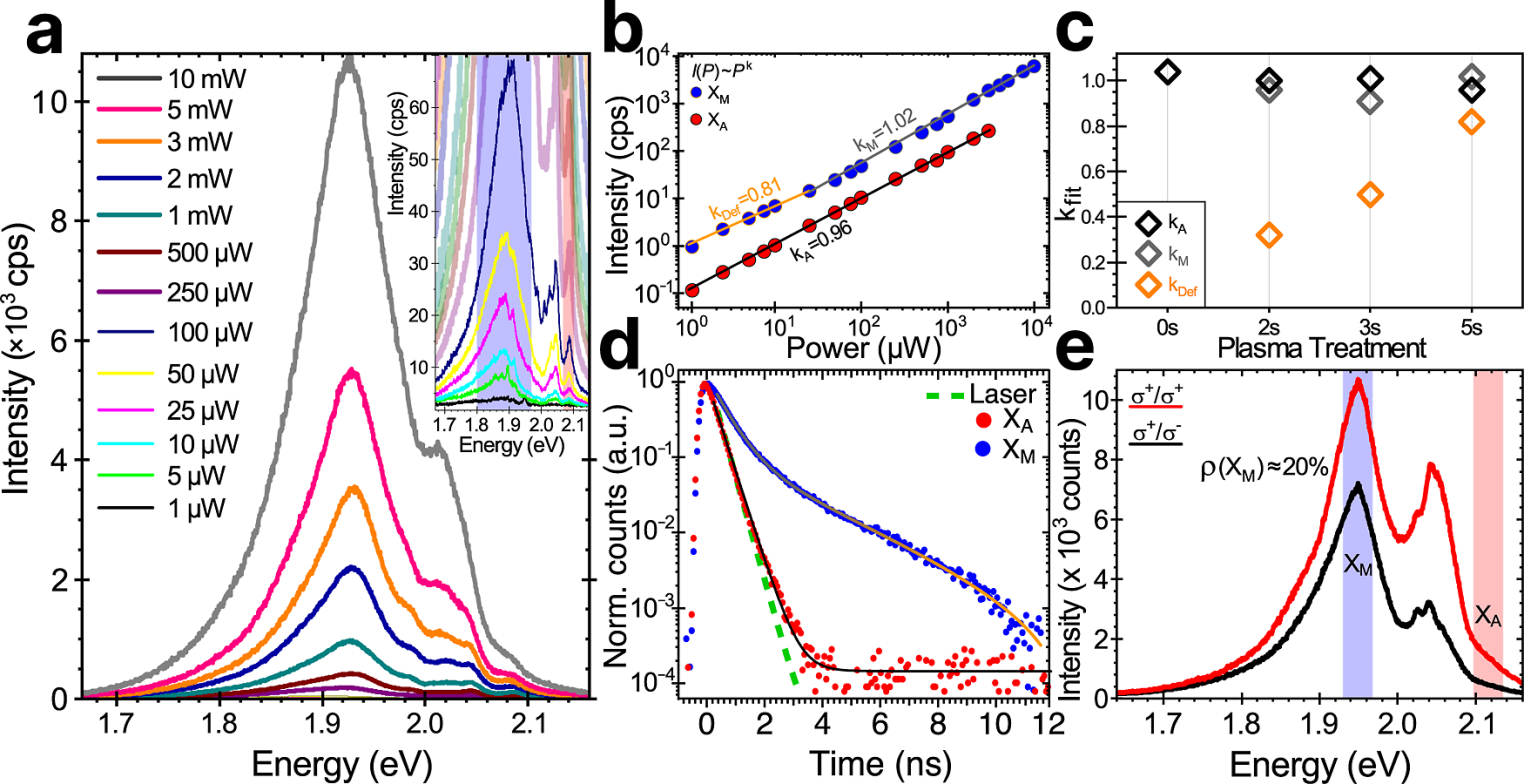
Optical characterization of the M band. (a) Emission spectra at increasing excitation laser power of a monolayer WS_2_ treated for 5 s at 25 W. The M band peak at ∼1.93 eV does not show any saturation behavior and dominates the spectrum at high power. Inset is a zoom of the spectral emission at low power. (b) PL intensity as a function of laser power for the A (red dots) and M (blue dots) excitons taken from the shaded windows of the inset in (a). Data are fitted with a power function *I*(*P*) = *aP*^*k*^. At low power, the fit (orange line) of low-energy emission indicates defect-localized emission of the A exciton; however, at higher powers (gray line), this is eclipsed by excitonic emission. (c) Summary of the power coefficients for the defect-localized (orange), M excitons (gray), and A excitons (black) for increasing plasma treatment time. (d) Comparison of the fluorescence lifetime of A (red dots) and M (blue dots) excitons. The lifetime of the A excitons on the order of a few picoseconds cannot be resolved with the time resolution of our setup, and its temporal response returns a lifetime comparable to that of the laser (green dashed curve). The fluorescence lifetime of the M band fits well with a double exponential function with τ_M_ = 680 ps and τ_Def_ = 3.4 ns. (e) Emission spectra of another 5 s plasma-treated WS_2_ sample taken at *T* = 8 K excited with σ^+^ circular polarization (with 500 μW laser excitation) and collected with σ^+^ (red line) and σ^–^ (black line).

Although the M resonance peak appears in the emission spectrum with an energy comparable to the one associated with the defective band,^28,29^ the former has a fundamentally different nature. Opposed to the defective band that stems from A excitons that, upon absorption of light, get bound to impurities or lattice defects, the emerging peak has a different origin, as it is an optical resonance of the material. We note that localized emission from A excitons bound to defects cannot be ignored in our samples due to the presence of vacancies as a consequence of the plasma treatment.^14,18,21^ To differentiate between the M band and the typical defective emission, we performed power-dependent PL measurements. In these measurements, the intensity of an optical feature’s emission can be fitted to a power function: *I*(*P*) = *aP*^*k*^, where the exponent, *k*, should follow the classification of the underlying radiative process. Exciton-like recombination is expected to exhibit a power law with *k* ≈ 1, recombination from bound states involving defects with *k* < 1, and from biexciton with *k* > 1.^30,31^ The power evolution of our pristine WS_2_ sample is shown in Supporting Information Figure S15. In Figure 4a, we report the evolution of the low-temperature emission spectrum for a 5 s-treated sample as a function of the laser power over 4 orders of magnitude. At high power, M emission does not saturate and becomes the dominant emission pathway for the photoexcited carriers. We extract the PL intensity of the spectra in the band from 2.07 to 2.10 eV to track the emission of the neutral A exciton and from 1.80 to 1.97 eV to track the lower energy features in the sample as a function of the pump power, shown in Figure 4b with red and blue circles, respectively. At low to medium-high powers where the neutral A exciton is apparent and not yet lost in the shoulder of trionic and biexcitonic emission, the fit returns *k*_A_ = 0.98 ± 0.02 for A excitons. The laser power density used in our experiments is in the range 10^1^–10^4^ W cm^–2^ (corresponding to a generation rate of 10^6^–10^10^ cm^–2^ ps^–1^, see Methods) and the observed linear behavior of the A exciton is compatible with previous reports considering the reduction of the exciton–exciton annihilation rate due to the partial encapsulation of the mixed-phase WS_2_.^32−34^ In the low energy range, we can distinguish two regimes. For pump powers at or below 25 μW, the fit returns *k*_Def_ = 0.81 ± 0.02, suggesting that this emission is dominated by defect-localized emission.^29^ At higher pump powers, the fit returns *k*_M_ = 1.02 ± 0.01, indicating that the predominant low energy emission is different from the defective band. This evidence suggests that this low-energy emission peak is associated with an excitonic transition. Further work should employ more sophisticated theoretical models, such as GW calculations, to investigate the excitonic nature of the M band. We additionally note that while the remaining 1H in the sample has defects, such as vacancies induced by the plasma treatment, the degree of defectiveness is not so extreme as the neutral exciton can still be individually resolved and is only moderately broadened by irradiation (fwhm of Gaussian fit of *X*_A_ increases from 11.0 ± 0.3 to 15.5 ± 1.1 meV with 5 s irradiation time, see Supporting Information Figure S16). We observe a reduction of the spectral weight of the A excitons with respect to the one of the M band when the treatment time is increased (Supporting Information Figure S17). This is expected as longer plasma irradiation leads to further growth of the 1T phase but decreases the remaining 1H content and also quenches the overall A emission through the formation of additional sulfur vacancies.^24^ The rise of the defect density as a function of the irradiation time is further confirmed by the growth of the saturation coefficient for the defective band (*k*_Def_ shown in Figure 4c). When we treat the sample further, the A exciton in the PL emission practically vanishes, and 1H and 1T Raman modes are very weak, indicating that the remaining 1H phase is of poor optical quality (Supporting Information Figure S18). However, we still see nonsaturating PL emission from the M band resonance. The nonsaturating PL emission ascribed to the M exciton is additionally observed in room temperature measurements (Supporting Information Figure S19), confirming the robustness of this optical transition. We note that in doped TMD monolayers, deviation of the emission intensity from the linear power-law similar to the one observed for the M band in Figure 4 could result from the interplay between neutral and charged excitons.^35^ However, this effect should not play a major role in our experiments as our samples are only moderately doped, as confirmed by the comparable emission of the A excitons and trions (Supporting Information Figure S15a). Nevertheless, the question of the presence of charged bound states at the M band is still open and will be the focus of further studies.

The emergent M band can be additionally distinguished from defect-localized emission through fluorescence lifetime measurements as illustrated in Figure 4d. While the short lifetime of the A exciton is of the order of few ps^36^ and cannot be resolved by our setup that has a detector-limited resolution of 400 ps, the fluorescence from the low energy peak can be measured as it has a much longer lifetime. The normalized temporal response from the low energy peak fits well with a double exponential function: . The fit returns τ_M_ = 680 ps, *a*_M_ = 0.75, τ_Def_ = 3.4 ns, and *a*_Def_ = 0.13, indicating the presence of two contributions to the low energy peak: a predominant contribution with a lifetime of 680 ps and a minor contribution with a longer lifetime of 3.4 ns. The latter is consistent with the recombination from localized excitons in WS_2_,^29^ and the small amplitude corroborates the hypothesis of a weak contribution of the defective band in the emission from the low energy peak. Circular polarization is a key property of excitons in TMDs that enables the study of many-body phenomena, and, at the same time, can be employed to differentiate exciton species, including free and localized A excitons.^37^ We measured the polarization-resolved PL emission spectra of treated WS_2_ (Figure 4e) excited with σ^+^ circular polarization and collected with σ^+^ (red line) and σ^–^ (black line). We measure a degree of polarization,  (where *I*^+^ and *I*^–^ are the emission intensity of σ^+^ and σ^–^ polarized light, respectively), for the M band of ∼20%. We find that the emission from the M band does not show any linear polarization (Supporting Information Figure S20). The circular dichroism of the M band could be explained by the contribution of the 1H conduction band edge at the K valley in the optical transition (Supporting Information Note 3 and Table S1). However, further investigation on the polarization properties, as well as the fine structure and gate dependence, of the M band should be performed on mixed-phase samples with individual structures that, as suggested by our theoretical calculations, generate single and narrow resonances, thus facilitating the photophysical characterization, which could be leveraged for photonic and optoelectronic applications. Such samples can be fabricated, for example, with direct growth of mixed-phase monolayers^11,12^ or deterministically generated in an electron microscope with the combination of temperature and electron dosage^18^ to produce 1T grains with enhanced control over the phase boundaries when compared to plasma irradiation procedures. Additionally, strain, temperature, and vacancy concentration have a clear influence on the energetic balance between TMD polymorphs and could be investigated as a means to engineer reversible pathways for the 1H/1T phase mixtures discussed in this work.^24,38−40^

## Conclusions

In summary, we present a scalable approach to generate additional optical resonances in the spectrum of the TMD systems. By taking advantage of the stable intermediate steps of the 1H → 1T phase transition process, we have theoretically and experimentally shown that atomic-sized mosaics of mixed-phase patches can be obtained from pristine 1H samples by triggering phase changes via irradiation with short bursts of plasma at mild power. The resulting atomic structures are robust and can be used in heterostructures and device assemblies. This atomic manipulation of the TMD lattice induces the formation of additional electronic bands that give rise to optical transitions in part of the spectrum that were previously out of reach. Moreover, the emergent transitions are characterized by useful optoelectronic and photonic properties, including a strong emission, long radiative lifetime, and circular polarization. This observation opens unexplored scenarios for light harvesting applications, active metamaterials, optoelectronics, and quantum materials.

## Methods

### Sample Preparation

Monolayers of WS_2_ were prepared by mechanical exfoliation of bulk crystals (HQ Graphene) with tape (Semiconductor Equipment Corp., “Blue Low Tack” tape) onto Polydimethylsiloxane (PDMS) films (Gel-Pak, Gel-Film). Monolayers were then transferred to Si/SiO_2_ substrates (University Wafer, ⟨100⟩ silicon wafer with 285 nm wet thermal oxide) under an optical microscope as part of a home-built transfer stage setup by pressing the PDMS film to the silicon wafer at 40 °C, heating to 70 °C to release the flake, and retreating the PDMS film. Protective capping layers of high-quality hBN, applied following plasma treatments, were fabricated by the same method and applied directly on top of the target WS_2_ flakes. After encapsulation, a brief annealing under an atmospheric-pressure argon environment at ∼200 °C is performed to improve interfacial quality between the WS_2_ and hBN flakes.

### Phase Change Plasma Treatment

Monolayers of WS_2_ were converted from the pristine 1H structure to a 1H/1T mixed phase by mild argon plasma treatment (Oxford Instruments, PlasmaProNPG80 RIE). Argon flowed at 30 s.c.c.m. into a chamber pressure of 50 mTorr, and then 25 W 13.56 MHz RF power was supplied for variable times from 2 to 10 s.

### Sample Characterizations

Samples for TEM characterizations were transferred by PPC (polypropylene carbonate) films to TEM grids (Ted Pella, UltrAuFoil). Characterization was done with a Cs-corrected FEI Titan Themis 200 S/TEM instrument at 80 kV acceleration voltage. Simulated TEM images were generated with clTEM.^41^ Raman spectroscopy was measured using a WITec alpha300R confocal Raman microscope using λ = 532 nm illumination. XPS characterizations (Physical Electronics, PHI-VersaProbe II) were conducted with a 2.5 W, 15 kV Al–Kα beam. XPS spectra were charge-corrected by calibrating the binding energy by indexing the adventitious carbon peak to 284.8 eV.

### Optical Characterizations

PL, reflectivity, and spectroscopic measurements were performed in a home-built confocal microscope setup coupled to a closed-cycle cryostat. Experiments were performed in a reflection geometry by exciting the sample with a continuous-wave green laser (532 nm) with a laser spot on the sample of radius 1 μm, a broadband tungsten halogen lamp peaked at λ ∼ 726 nm (Ocean Insights, HL-2000), or a supercontinuum pulsed laser (SuperK FIANIUM) with a tunable filter with a bandwidth of 2 nm. PL measurements were performed using avalanche photodiodes (APDs) (Excelitas, SPCM-AQRH-14) with a ∼400 ps time resolution. The laser reflection was removed from the PL by long-pass filters. Spectra were measured by a spectrometer with a 150 G/mm grating and an EMCCD camera. All cryogenic measurements were taken at a temperature of 8 K ± 1 K depending on the base temperature of the cryostat, where the temperature-driven shift of the A exciton over this range (±1 K) should be negligible (∼0.06 meV).^42^ Measurement of fluorescence lifetime was conducted with the tunable filter of the supercontinuum laser set to λ = 532 nm and an applied power of 100 μW. The generation rate was estimated considering an efficiency of the objective lens of 90% and an absorption for WS_2_ at the energy of the green laser of 5%.^43^

### Theoretical Calculations

The structural geometry relaxations and the electronic structure calculations are performed using density-functional theory utilizing the Vienna Ab initio Simulation Package (VASP) (version 6.3.2) with the PBE exchange-correlation functional^44,45^ and SOC. To construct the mixed phase structures, we started with expanding a primitive cell of monolayer 1H-WS_2_ with a lattice constant of 3.18 Å to a (unless noted otherwise) 9 × 9 × 1 supercell. We used a vacuum space of 14.17 Å perpendicular to the WS_2_ monolayer. We used the pristine 1H structures as the starting point; then we introduced grains of the 1T phase with varying geometries by displacing select sulfur atoms in one of the chalcogen planes to 1T-like positions. To preserve the structural stability of the mixed-phase supercells, vacancies were introduced on the same sulfur plane next to the newly created 1T grain. We optimized the structures by relaxing the atom positions until forces were less than 10–4 eV nm^–1^ with a plane wave energy cutoff of 420 eV. The electronic band structure of 1H/1T supercells were found through nonself consistent calculations along a *k*-path of M → K→ Γ → M in the Brillouin zone with 30 *k*-points between these high symmetry locations. Optical spectra calculations were used to simulate the dielectric constant with the aid of Sumo.^46^ We used the VASP Band Unfolding package (https://github.com/QijingZheng/VaspBandUnfolding) to unfold the band structure of the supercell as well as for calculations of transition dipole moments and transition densities between the states. Atomic structures of supercells were visualized using VESTA.^47^

## Data Availability

All data are available in the main text or the Supporting Information. The data sets generated during and/or analyzed during the current study are available from the corresponding author on request.

## Supporting Information Available

Additional materials characterizations; extended DFT analysis, further optical characterizations, side and top views of 1T and 1H phase WS_2_, lower magnification TEM images of treated samples, comparison of experimentally observed linear 1T grains with 1T-L1 and 1T-L2 structures, low-magnification TEM images and the corresponding diffraction patterns, decomposition of Raman spectra by multipeak Lorentzian fitting, XPS spectra, AFM maps of the WS_2_ monolayer before and after RIE treatment, orbitals of the conduction band states at the K valley that generate *X*_A_ in pristine 9 × 9 × 1 1H-WS_2_, orbitals of the *X*_M_ transition in the 1T-L1 structure calculated in a 9 × 9 × 1 1H-WS_2_ supercell, DFT results from pristine WS_2_, transition density of *X*_A_ for 1T-Tr2 in a 9 × 9 × 1 1H-WS_2_ supercell, atomic structure of 1H/1T supercells used for calculations, atomic model of the two types of zigzag interfaces between 1T and 1H phases, DFT results of 1T-Tr2 in 9 × 9 × 1 1H-WS_2_ with alternate orientation, power-dependent PL spectra of pristine WS_2_, effect of plasma on strength of optical resonances, power-dependent PL spectra for RIE treatments of varying times, characterization of samples treated at 25 W for 10 s, room temperature power-dependent PL spectra for treated WS_2_, linear polarization of treated sample, and degree of spin polarization of orbitals responsible for M band resonances (PDF)
