# Supplementary material for: Emergent Optical Resonances in Atomically Phase-Patterned Semiconducting Monolayers of WS2
Source: ACS Photonics. 2024 Aug 16;11(9):3784–93. doi: 10.1021/acsphotonics.4c00983 (PMC11413843; doi:10.1021/acsphotonics.4c00983)
Supplement: Supplementary file 1 — ph4c00983_si_001.pdf [file ph4c00983_si_001.pdf]

Supplementary Information for

Emergent optical resonances in atomically phase-patterned semiconducting monolayers of WS_2

*John M. Woods[1 †], Saroj B. Chand[1 †], Enrique Mejia[1], Ashok Adhikari[1], Takashi Taniguchi[2], Kenji Watanabe[3], Johannes Flick[4,5,6], Gabriele Grosso[1,6]**

1. Photonics Initiative, Advanced Science Research Center, City University of New York, New York, NY, USA

2. International Center for Materials Nanoarchitectonics, National Institute for Materials Science, 1-1 Namiki, Tsukuba 305-0044, Japan

3. Research Center for Functional Materials, National Institute for Materials Science, 1-1 Namiki, Tsukuba 305-0044, Japan

4. Center for Computational Quantum Physics, Flatiron Institute, New York, NY 10010, USA

5. Department of Physics, City College of New York, New York, NY 10031, USA

6. Physics Program, Graduate Center, City University of New York, New York 10016, New York, USA

† Equal contribution

* Corresponding author

E-mail: *ggrosso@gc.cuny.edu*

Contents of Supplemental Information: 19 pages, 20 figures, and 1 table

Supplementary Note 1 - Additional Materials Characterizations

The atomic structure of 1H and 1T phases of WS_2 is shown in **Supplementary Figure S1**. To determine the atomic structure of the linear 1T grains seen in the TEM images of plasma treated WS_2 samples, we compare simulated TEM images for 1T grains that are one or two lattice vectors wide, 1T-L1 and 1T-L2 respectively, with the experimental images in **Supplementary Figure S2**. We find that the linear structures in our experimental structures match with a 1T-L1 structure. **Supplementary Figure S3** shows additional 1T grains from a zoomed out HR-TEM image of the 5s- and 10s-treated samples shown in **Figure 1** of the main text. Selected area diffraction (SAD) patterns taken from large areas of treated WS_2 samples (**Supplementary Figure S4**) maintained a 6-fold symmetry indicating that the newly generated phase with treatment is the 1T phase and that there exist no high-angle grain boundaries between regions of 1H and 1T. Raman spectroscopy (**Supplementary Figure S5**) of the treated samples features the appearance of peaks associated with the 1T-WS_2 phase in addition to the Raman modes of 1H-WS_2.[1–3] In this sample the decomposition of 1H peaks with a multi-peak fitting procedure returns a slight broadening of the Raman peaks which is consistent with the addition of disorder to the system in the form of vacancies and 1H/1T grain boundaries. X-ray photoelectron spectroscopy (XPS) in **Supplementary Figure S6** finds a shift to lower binding energy of the W-4f orbitals 1T samples that is consistent with previous reports.[4–6] In the lightly plasma treated WS_2 sample, the W-4f peaks are broadened indicating a co-presence of 1H and 1T phases in the sample. When comparing WS_2 flakes before and after treatment, we find that the surface roughness extracted from AFM measurements does not significantly change with plasma irradiation (**Supplementary Figure S7**).

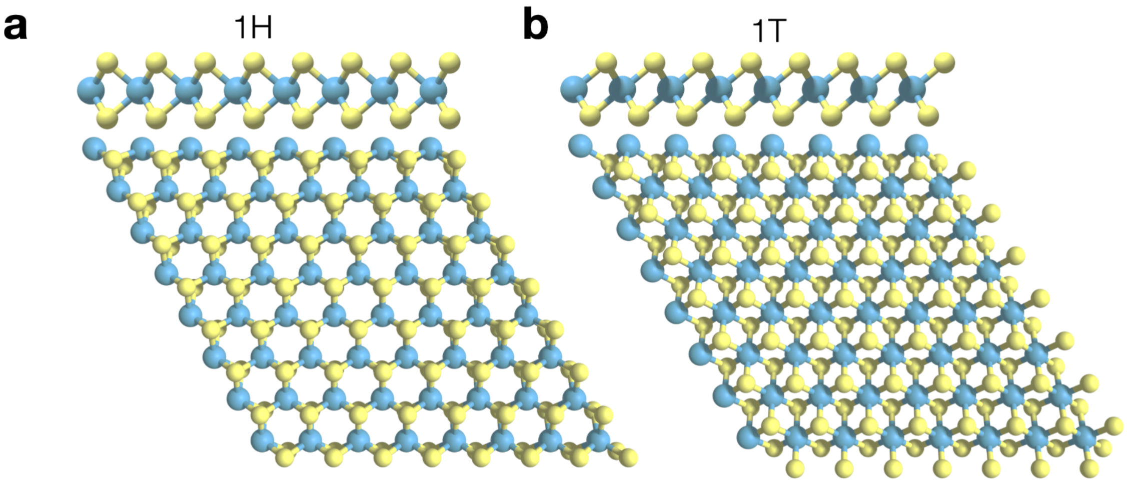

Supplementary Figure S1. Side and top views of 1T (**a**) and 1H (**b**) phase WS_2.

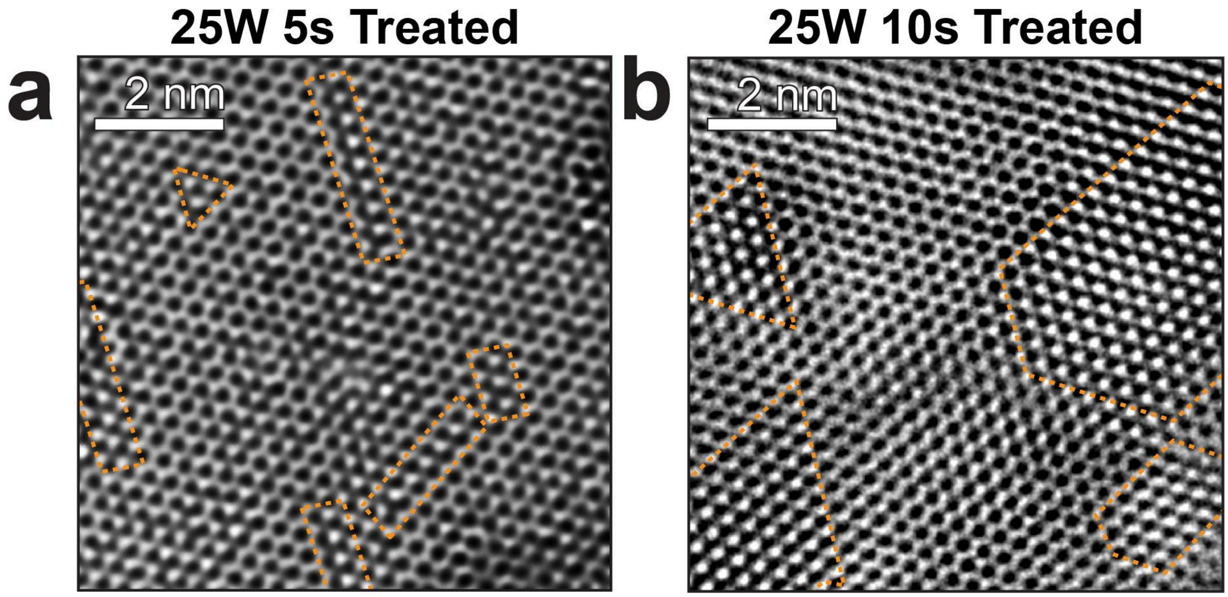

Supplementary Figure S2. Lower magnification TEM images of the (**a**) 5s- and (**b**) 10s-treated samples featured in **Figure 1** of the main text showing further examples of 1T gains with linear, triangular, or polygonal morphologies (highlighted with dashed orange lines).

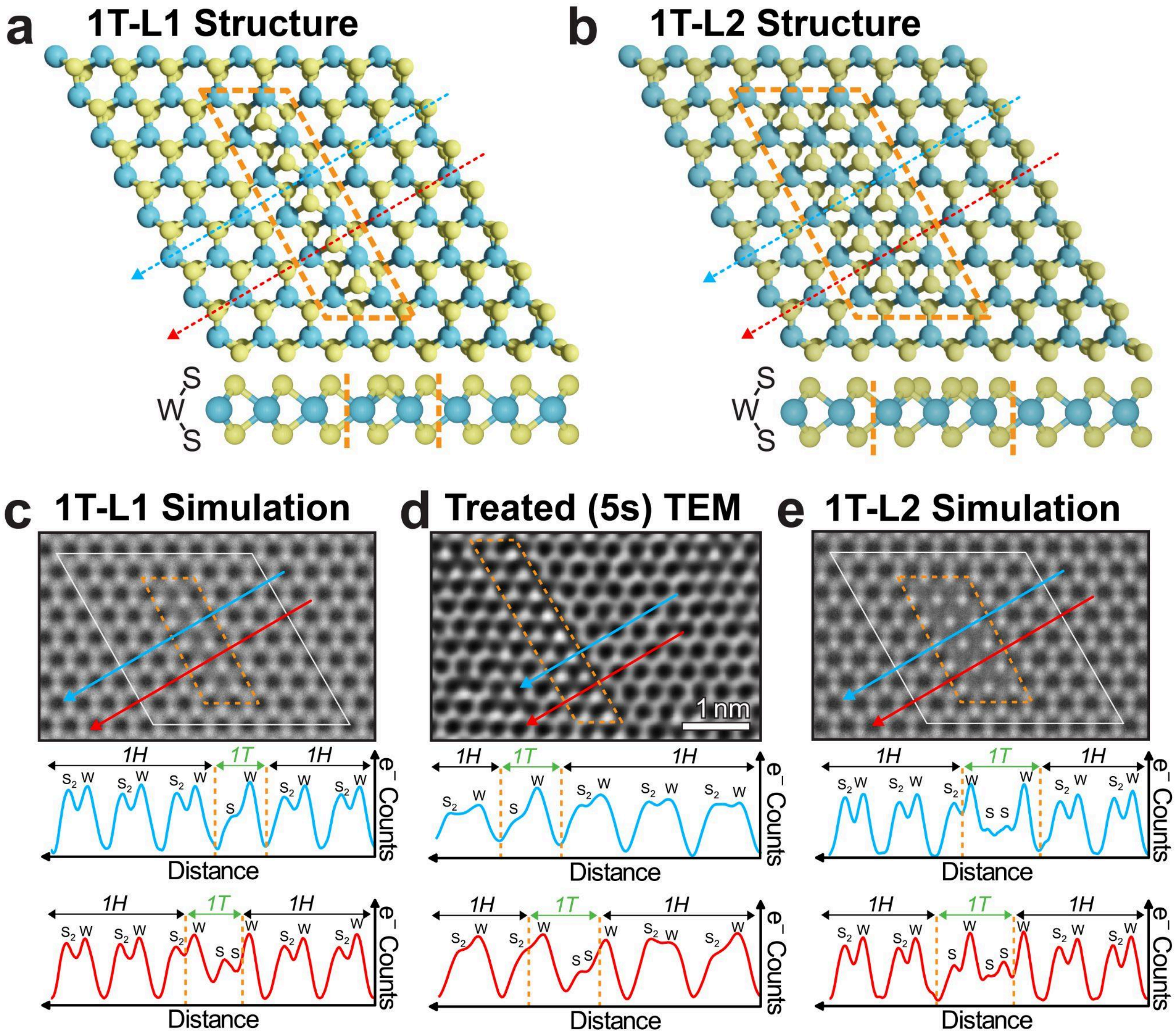

Supplementary Figure S3. Comparison of experimentally observed linear 1T grains with 1T-L1 (**a**) and 1T-L2 (**b**) structures. There are two inequivalent paths which cut through the 1T grain shown with blue and red arrows. These two line profiles are taken along these paths in simulated images of 1T-L1 (**c**) and 1T-L2 (**e**) structures to compare with experimentally observed linear 1T grains in the 5s-treated sample (**d**). We find that the images and line profiles of the plasma treated sample are most consistent with the 1T-L1 mixed phase structure.

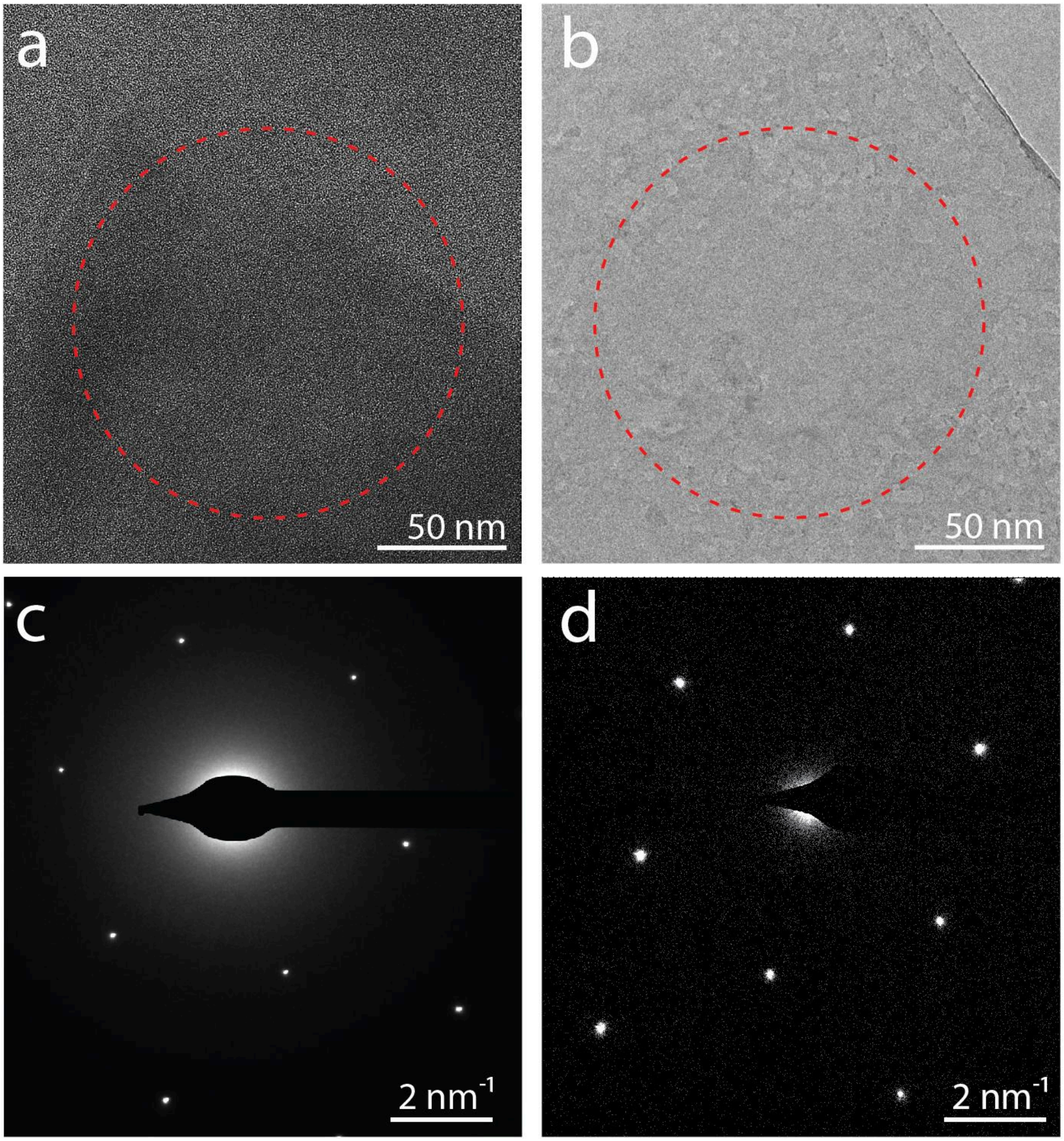

Supplementary Figure S4. Low-magnification TEM images and the corresponding diffraction patterns collected with a selected area aperture (location indicated with dashed red line) of pristine (**a**, **c**) and 25 W 10s RIE treated (**b**, **d**) WS_2. The 6-fold symmetric diffraction pattern is indicative of both 1H and 1T phases of WS_2.

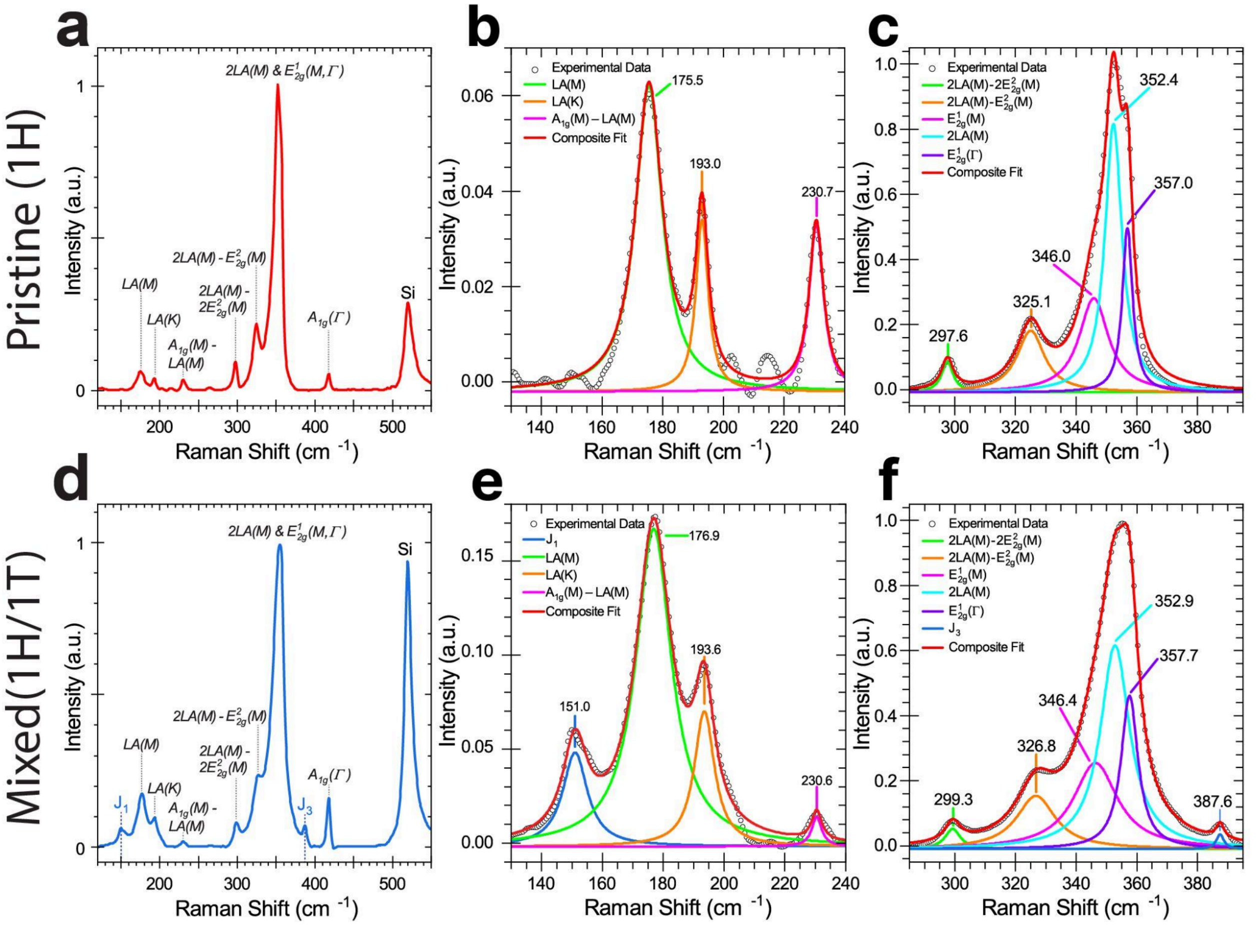

Supplementary Figure S5. Decomposition of Raman spectra by multipeak Lorentzian fitting. Spectra from pristine (**a**, **c**) and 25 W, 5 s plasma treated 1H/1T mixed phase WS_2 samples (**b**, **d**) across the region where the J1 (**d-e**) and J3 (**d-f**) peaks appear in the plasma treated samples indicating the formation of the 1T phase. In addition to the appearance of the 1T Raman modes, we see that 1H Raman modes in the treated samples show a small amount of broadening which is consistent with the increased disorder from the formation of vacancy defects with plasma treatment.

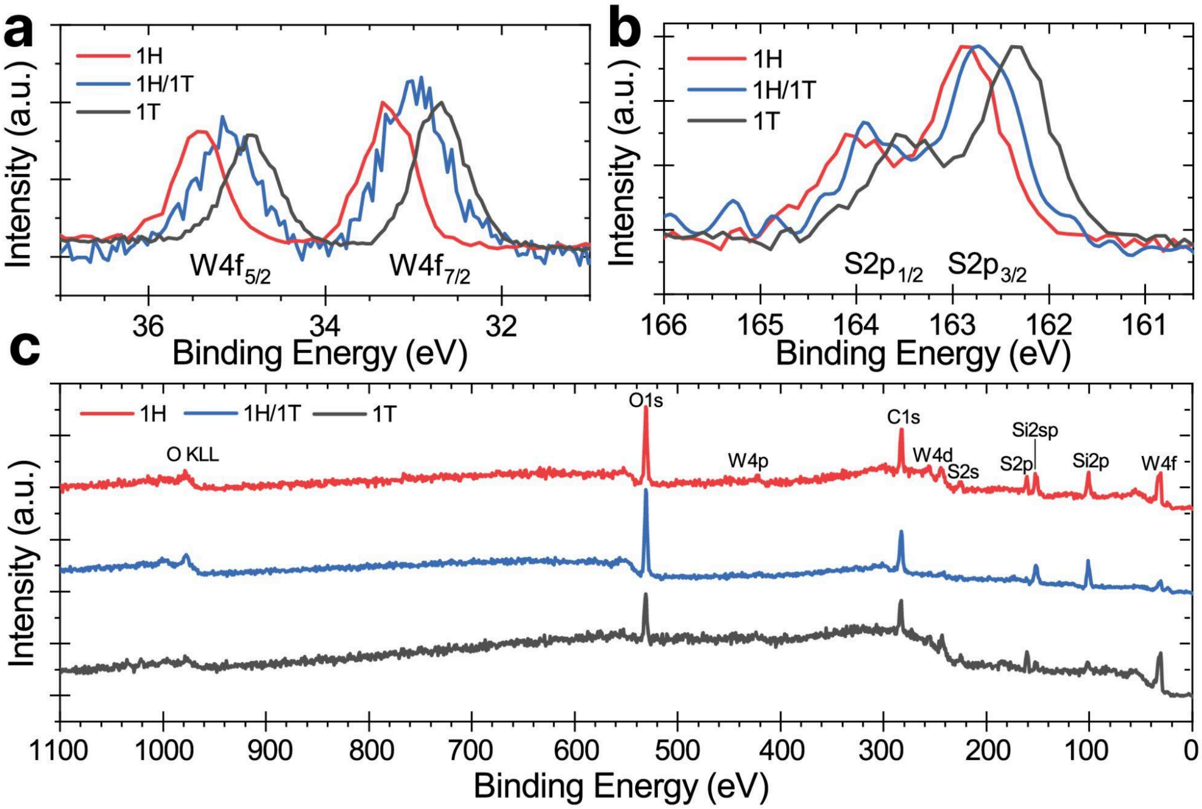

Supplementary Figure S6. XPS spectra of pristine 1H (red), fully 1T (black), and lightly plasma treated mixed phase WS_2 (blue). (a) The shift of the W-4f peak between 1H and 1T is comparable to literature reports.[4–6] Owing to the co-presence of 1H and 1T phases in the lightly plasma treated sample, the W-4f peak is broadened and falls between 1H-like and 1T-like energies. The S-2p peaks (b), while noisier, show a similar trend. The full-range survey scan shows no unexpected constituent elements. All XPS spectra were corrected by indexing the adventitious carbon peak to E=284.8 eV.

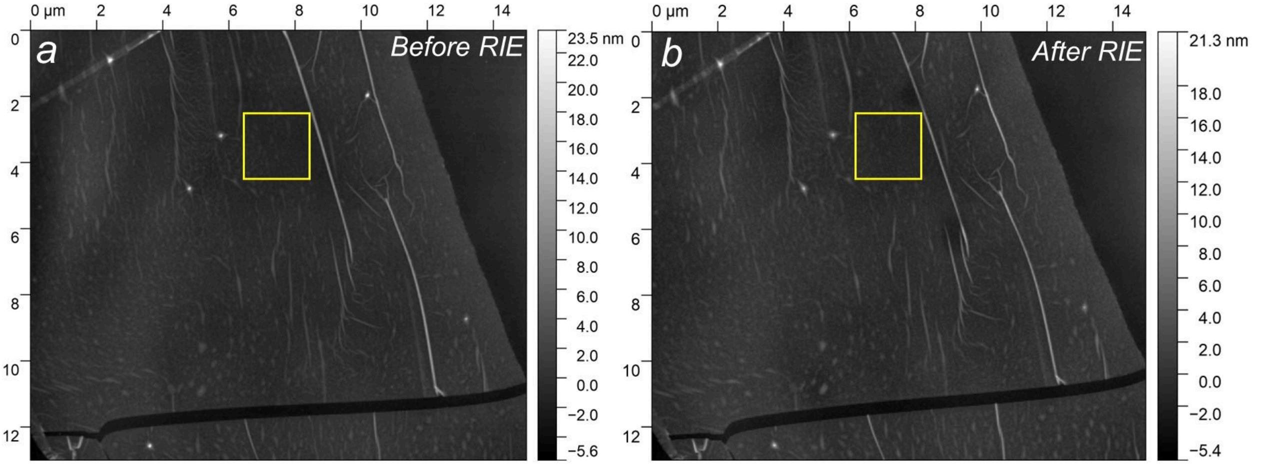

Supplementary Figure S7. AFM maps of WS_2 monolayer before (**a**) and after (**b**) RIE treatment at 25 W for 5 s. Calculation of the Root Mean Square (RMS) surface roughness (over the 2x2 µm box indicated in yellow) yields R_q = 75.0 pm before RIE and R_q = 76.9 pm after RIE indicating no significant increase of the surface roughness following treatment.

Supplementary Note 2 - Extended DFT Analysis

Additional density functional theory (DFT) calculations, supporting those reported in **Figure 2** and **Figure 3** of the main text, are reported below. All calculations are performed including spin orbit coupling. In pristine 1H the spatial distribution of conduction band orbitals (or Kohn-sham wave functions) associated with the *A* exciton (**Supplementary Figure S8**) are uniformly distributed and match the morphology of the conduction band orbital of our mixed phase supercells which participate in both X_A and X_M transitions. **Figure 2** of the main text displays the valence and conduction orbital states for X_M in the 1T-Tr2 supercell; the equivalent orbitals are shown for 1T-L1 (**Supplementary Figure S9**). For reasons covered in the discussion about **Figure 3e** of the main text, we find that in the 1T-L1 structure there are two strong resonances resulting from the linear 1T grain having two inequivalent interfaces. The occupied midgap states we see in the mixed-phase supercells are not present in pristine 1H, pristine 1T, or 1H supercells with a line of vacancies (**Figure S10**), indicating that the states responsible for the *M* band are specific to small grains of 1T and the interfaces these form with the surrounding 1H matrix. In contrast to the X_M transition density reported in **Figure 3a** of the main text which is concentrated around the 1H/1T interface, the transition density of X_A (**Figure S11**) is delocalized across the entire 1H region of the supercell. The supercells used in the calculation of data in **Figure 3e** of the main text are shown in **Figure S12**. **Figure S13** highlights the two types of "zig-zag" interfaces that can form between 1H and 1T phases. 1T-Tr*N* structures can feature only one type of interface, whereas the 1T-L1 structures will have "Interface-I" on one edge with 1H and "Interface-II" on the other. The analysis of the (Interface-I) 1T-Tr2 supercell in **Figure 2** of the main text is repeated in **Figure S14** for an "Interface-II" 1T-Tr2 supercell. The X_M resonance appears at a smaller detuning energy for this

structure, and this is consistent with what is seen for 1T-Tr*N* supercells more generally in **Figure 3e** of the main text. Displayed atomic structures of supercells in the following figures are the relaxed structures, indicating the mechanical stability of the analyzed supercells.

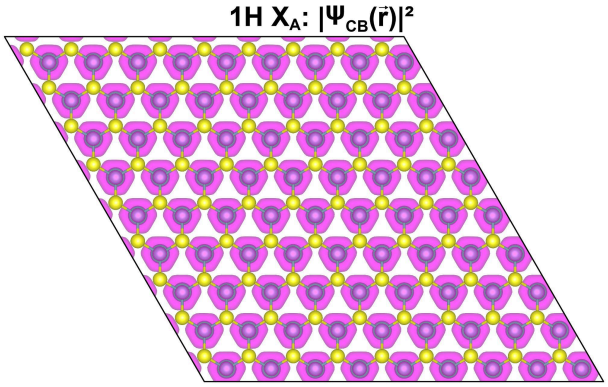

Supplementary Figure S8. Orbitals of the conduction band states at the K valley that generate X_A in pristine 9x9x1 1H-WS_2.

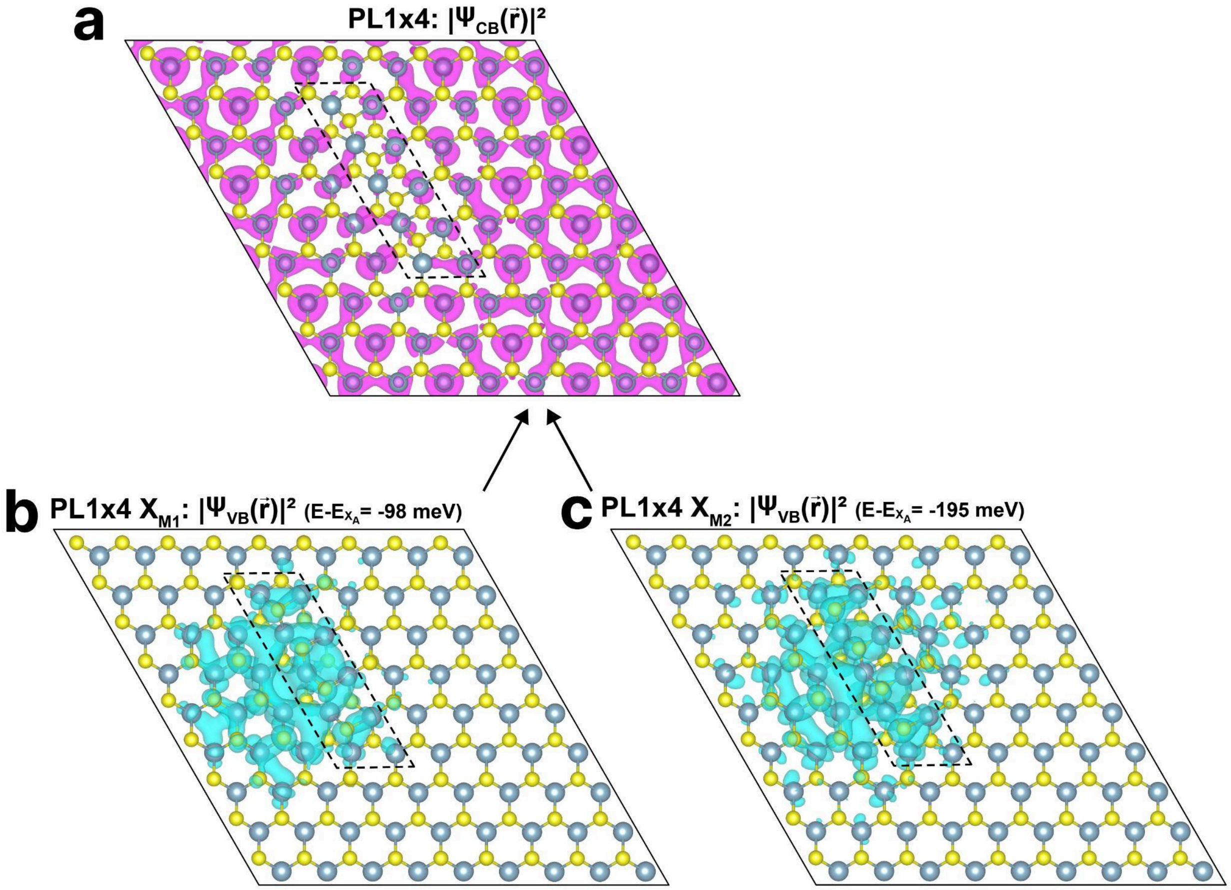

Supplementary Figure S9. Orbitals of the X_M transition in the 1T-L1 structure calculated in 9x9x1 1H-WS_2 supercell.

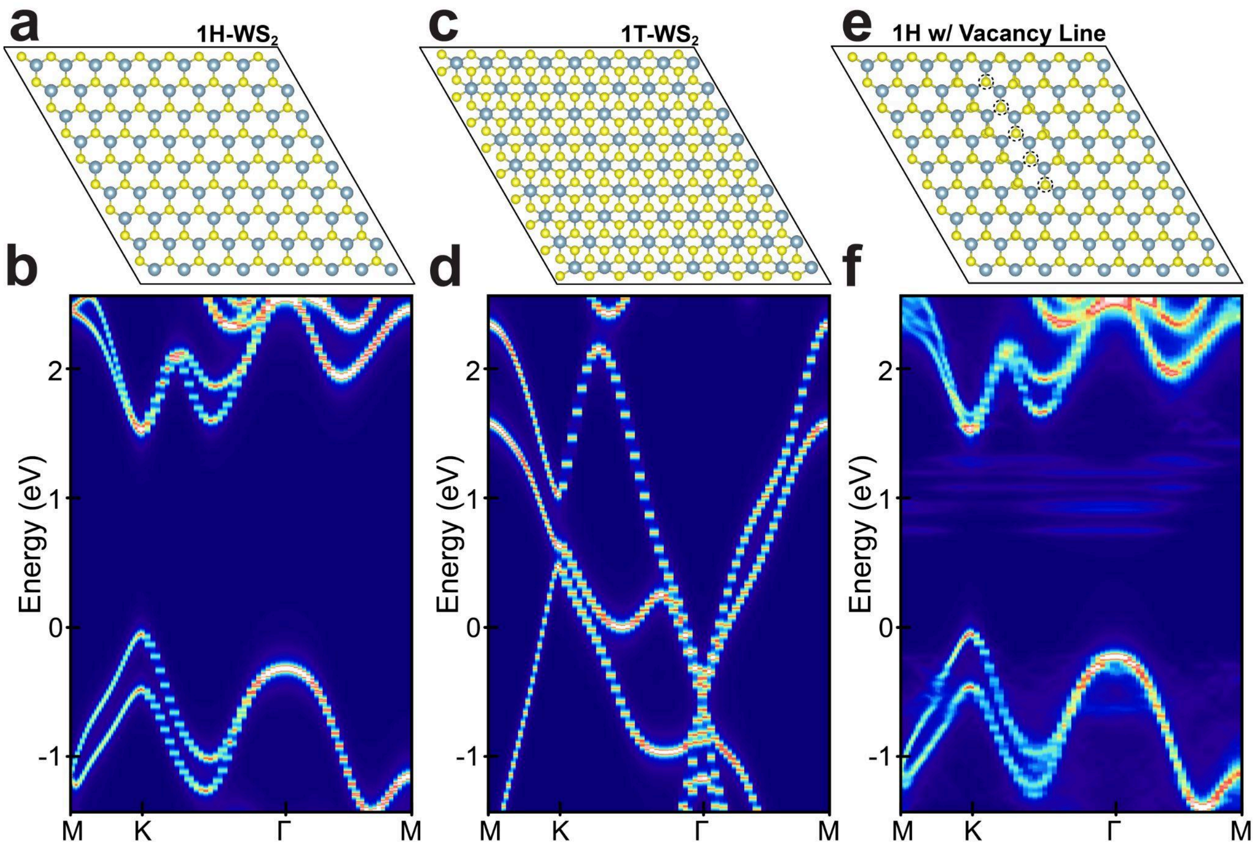

Supplementary Figure S10. DFT results from pristine 1H (**a**-**b**), pristine 1T (**c**-**d**), and 1H WS_2 with a line of vacancies highlighted with black, dashed circles (**e**-**f**). In the vacancy line structure, the only midgap states generated reside in the upper half of the band structure above the Fermi energy, and are thus unoccupied.

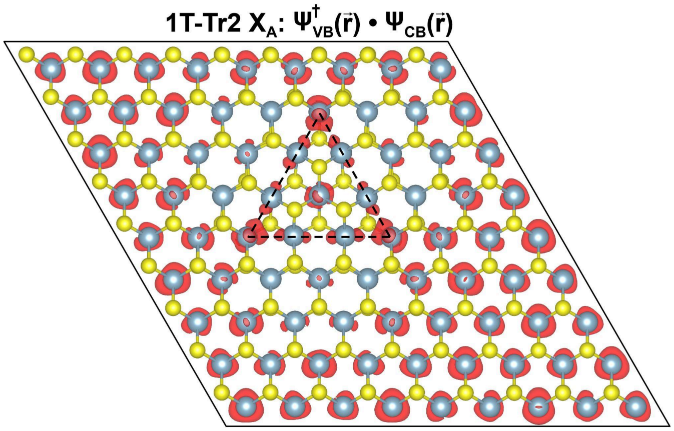

Supplementary Figure S11. Transition density of X_A for 1T-Tr2 in 9x9x1 1H-WS_2 supercell. The transition is delocalized across the 1H regions of the supercell.

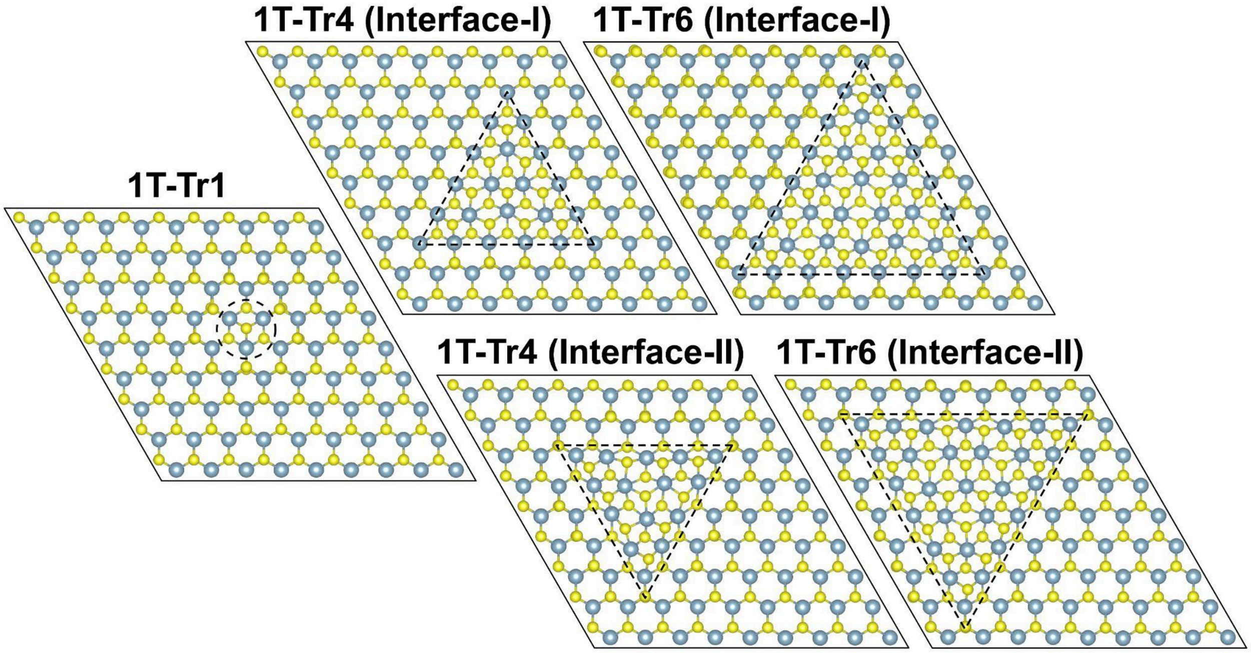

Supplementary Figure S12. Atomic structure of 1H/1T supercells used for calculations in **Figure 3e** of the main text. Structures of 1T-Tr2 are shown in **Figure 2c** and **Figure S14a**. Structure of 1T-L1 is shown in **Figure 2a**.

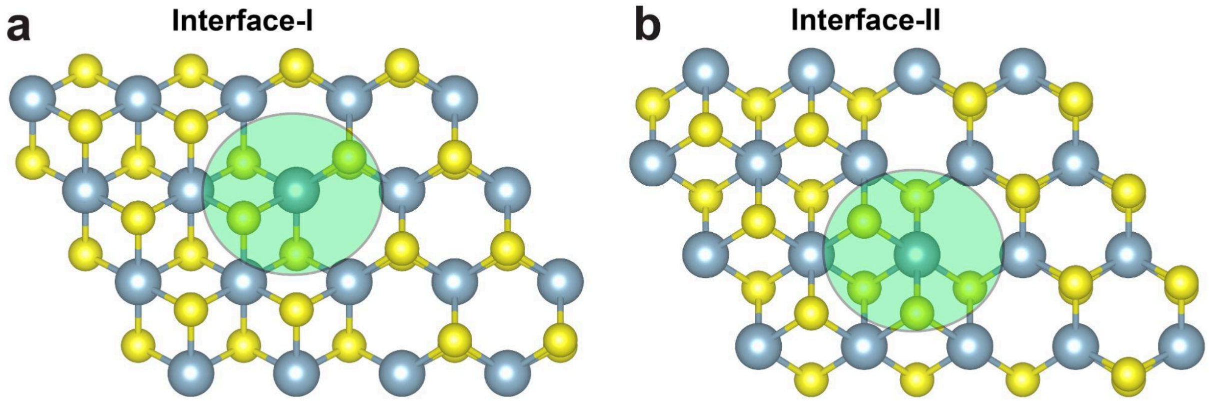

Supplementary Figure S13. Atomic model of the two types of zig-zag interfaces between 1T and 1H phases. View is rotated a few degrees off from the c-axis to make apparent where S_2 stacks are present in the structure. The coordination of sulfurs around tungsten atoms at the 1T/1H border are highlighted by green, shaded ellipses.

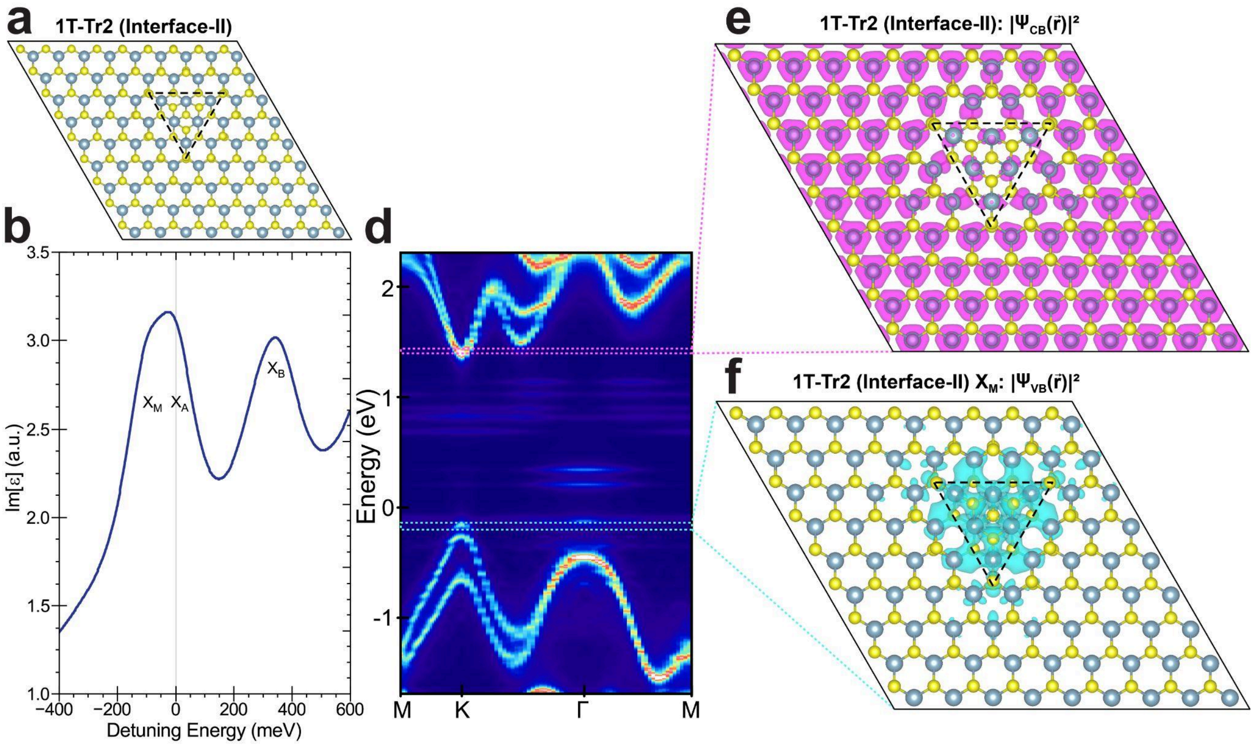

Supplementary Figure S14. DFT results of 1T-Tr2 in 9x9x1 1H-WS_2 with alternate orientation and resulting bond configuration (Interface-II) at the border with the 1H matrix.

Supplementary Note 3 - Further optical characterizations

In the spectra of pristine 1H-WS_2 (**Supplementary Figure S15a**) at cryogenic temperatures taken at low incident laser power, the *A* exciton and its usual complexes can be resolved. These complexes include in descending energy: (2.081 eV) the neutral exciton (X_A), (2.051 eV) triplet and singlet trions (X^-), and (2.026 eV) charged biexcitons (XX^-). The lower energy peaks (1.999 eV and 1.977 eV) have been previously attributed to localized bands.[7,8] However, more recent experiments in high-quality encapsulated WS_2 and WSe_2 show that the origin of these peaks is associated with dark excitons, in particular the T_1 exciton and the phonon replicas of the dark trions.[9–11] Exfoliated 1H-WS_2 monolayers are typically n-doped, and their high-power spectrum is dominated by emission from negatively charged biexciton whose intensity scales superlinearly as a function of pump power.[8,10] **Supplementary Figure S15b** reports the integrated intensity as a function of power for the energy range of the neutral exciton and charged biexciton in pristine 1H WS_2. As expected, the power dependence of neutral X_A emission can be fit with an exponent of $k \approx 1$ and at higher power, where it eclipses the complexes which exist in a similar energy range, biexcitonic emission grows with an exponent of $k > 1$ as expected. Although vacancy generation is inherent to the plasma treatment process, the remaining 1H regions of WS_2 nanoflakes after irradiation is still high-enough quality that the neutral *A* exciton peak is resolvable at low power, and, as reported in **Supplementary Figure S16,** only broadens from a FWHM of 11.0±0.3 meV for pristine to 15.5±0.3 meV for the 5s treated sample featured in **Figure 4** of the main text when comparing PL

spectra collected at equivalent incident laser powers. Power dependent PL spectra for 2s-, 3s-, and 5s-treated samples are compared in **Supplementary Figure S17**. We see that the *M* band grows increasingly dominant in the emission spectra as the plasma treatment time is increased. In the power fit of this data, we observe an increase of the sub-linear exponent of the power-dependent defect localized emission from k=0.32 to k=0.82. Moreover, the crossover power where X_M emission outpaces defect-localized emissions increases with plasma treatment time. Both trends are consistent with the increased density of vacancies in the remaining 1H regions of the flake. When the treatment time is set to 10s (**Supplementary Figure S18**), there is still non-saturating emission, but the *A* exciton complexes are no longer easily resolvable. Additionally, the Raman spectra for this sample has greatly reduced intensity. Furthermore, the robustness of the *M* band resonance is supported by the non-saturating emission at room temperatures for samples treated up to 5s with 25W plasma (**Supplementary Figure S19**). The *M* band emission doesn't show any linear polarization (**Supplementary Figure S20**).

If we consider the orbitals emerging from the DFT calculations that are related to the *M* band, it is not so surprising that the emission shows the modest circular polarization that it does in Figure 4e. The conduction band orbitals exist at the K points for both 1H and 1T phases, so even when hybridized at the interface, the chirality could be expected to remain for the 1H/1T supercells. In the analysis of the transition dipole moments composing the M band, we see that the strength of the dipole moment primarily occurs at the K or K' points for alternating spin states. To simulate how polarized we expect emission resulting from this band structure, we repeat the optical DFT calculations where we have artificially flipped the spin character of the unoccupied conduction band states. We can then compare an estimated chirality by comparing the dipole strength in the true and artificially spin flipped states. For pristine 1H we find X_A and X_B to be extremely polarized (>99.9999999%) as expected for the spin-separated valence and conduction bands. For X_M this chirality varies but it is >94% for 1T-L1 and both 1T-Tr2 supercells as shown in **Supplemental Table S1**. In actual measurements there are intervalley scattering and other many-body effects that cannot be gauged with these DFT calculations, but the DFT calculations are directionally in agreement with the result that the M band, as shown in Figure 4e, is circularly polarized but to a slightly lower level than the A-exciton emission.

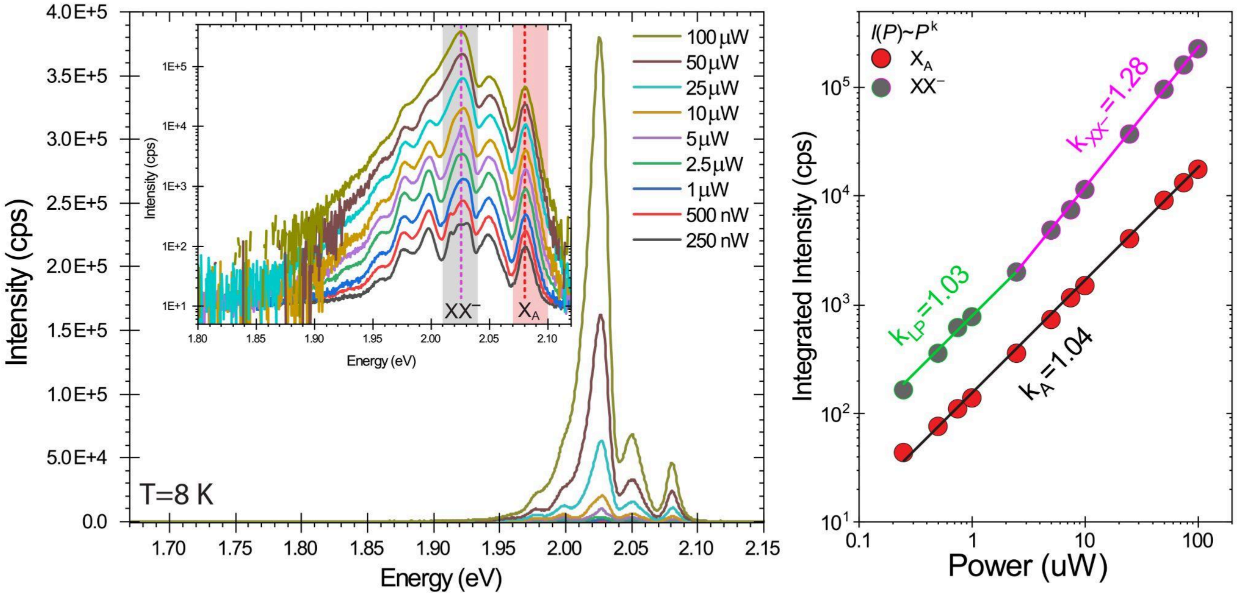

Supplementary Figure S15. (**a**) Power dependent PL spectra of pristine WS_2 taken at T=8 K. Inset shows the same plot in Log-scale to better visualize spectra at low power excitation There is no prominent emission at low-energies for E < 1.95 eV. Vertical lines indicate the location of the neutral *A* exciton (X_A) and the negatively charged biexciton (XX-). Lower energy complexes have been previously attributed to localized bands, but recent experiments in high-quality encapsulated WS_2 and WSe_2 suggest that these peaks may be related to dark excitons and phonon replicas of dark trions.[9,10] (**b**) Log-Log plot of PL intensity of the spectra from 2.069-2.100 eV (labeled X_A, red) and from 2.01-2.039 eV (labeled XX^-, gray) The plots are fit to the power curve, $I(P)=aP^k$. The neutral *A* exciton scales with k_A=1.04, and at high power the biexciton scales with k_{XX-}=1.28. At lower power emission in the window for the biexciton can be associated with phonon replicas and dark trionic emission related to the *A* exciton, and when fit returns k_{LP}=1.03

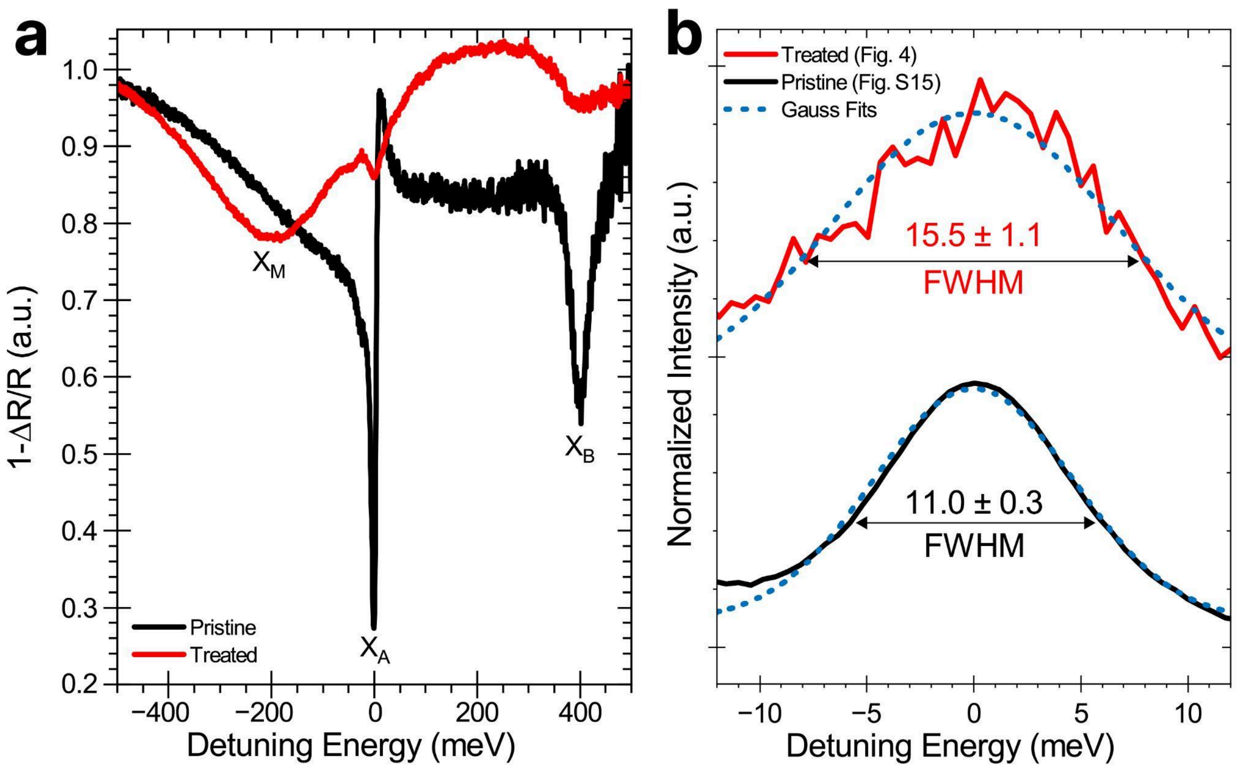

Supplementary Figure S16. (**a**) Change to absorption profile with plasma treatment. With treatment, absorption channels from 1H-phase WS_2 (i.e. X_A & X_B) are diminished in strength, but are still present. This is consistent with the decrease in 1H coverage (as it is converted to 1T) and quenching effects from the metallic 1T phase and the excess vacancies which are a byproduct of the plasma treatment. The *M* band absorption in the treated sample is stronger in magnitude than the baseline lower-energy absorption that is present in pristine samples. Data is taken from the scans displayed in **Figure 2h**. (**b**) Broadening of neutral *A* exciton with treatment. Treated spectrum is the one taken with a power of 100 μW from the scan in **Figure 4** of the main text. Pristine spectrum is the one at 100 μW from the power scan in **Figure S15**. Spectra are plotted with a vertical offset for the sake of clarity. The FWHM of the neutral *A* exciton peak grows from 11.0±0.3 meV to 15.5±1.1 meV. The broadening is expected due to the aforementioned detrimental effects of the plasma treatment, but it is still relatively sharp indicating that the remaining 1H regions are still of good optical quality.

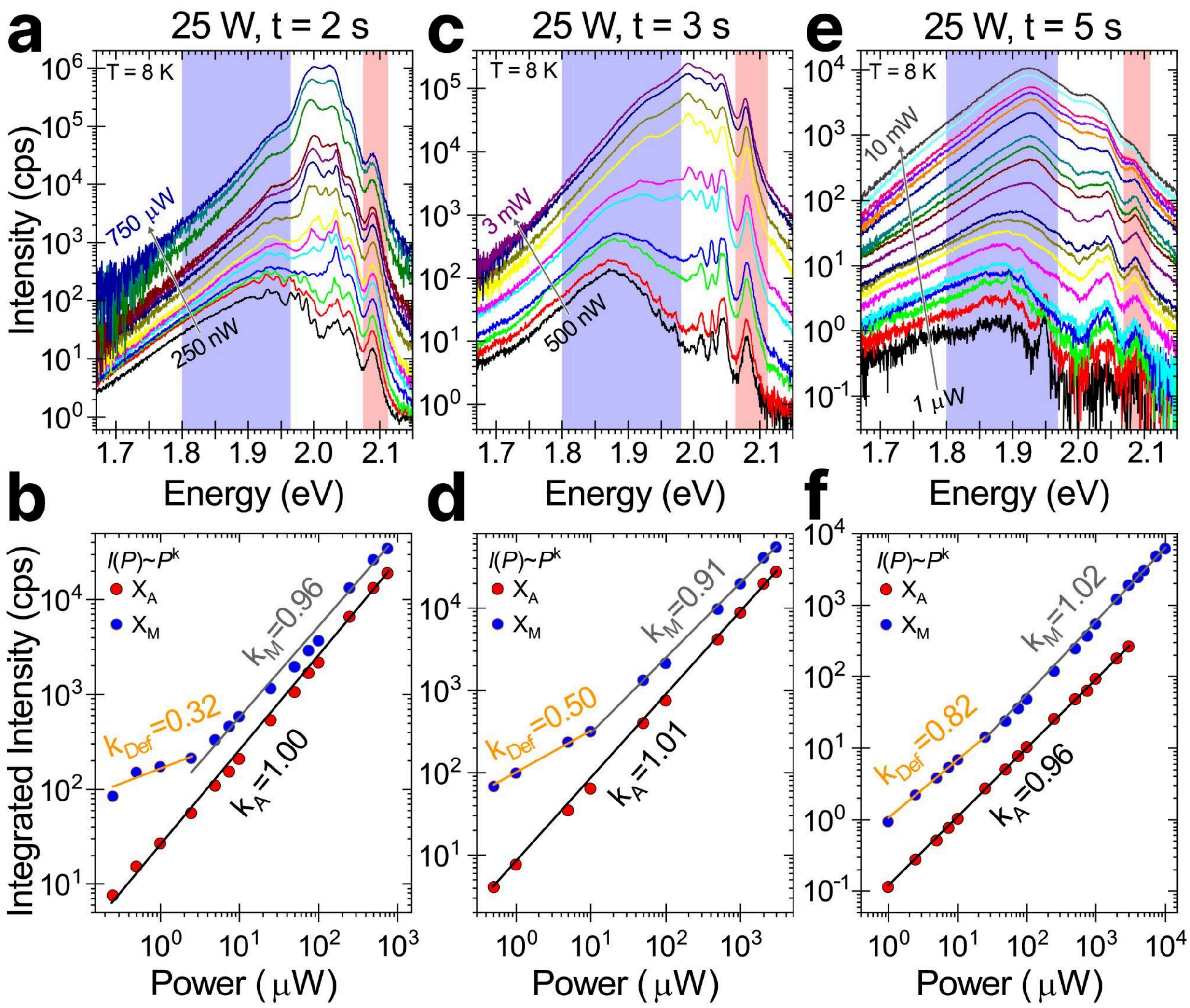

Supplementary Figure S17. Power dependent PL spectra for 25 W RIE treatments of varying time: 2 s (**a**), 3 s (**c**), and 5 s (**e**, reproduced from **Figure 4**a in log scale). All spectra were taken at T=8 K. As plasma treatment time is reduced, the *M* band emission (E≈1.93 eV), while present in the signal, no longer eclipses the negatively charged biexciton emission (E≈2.02 eV).

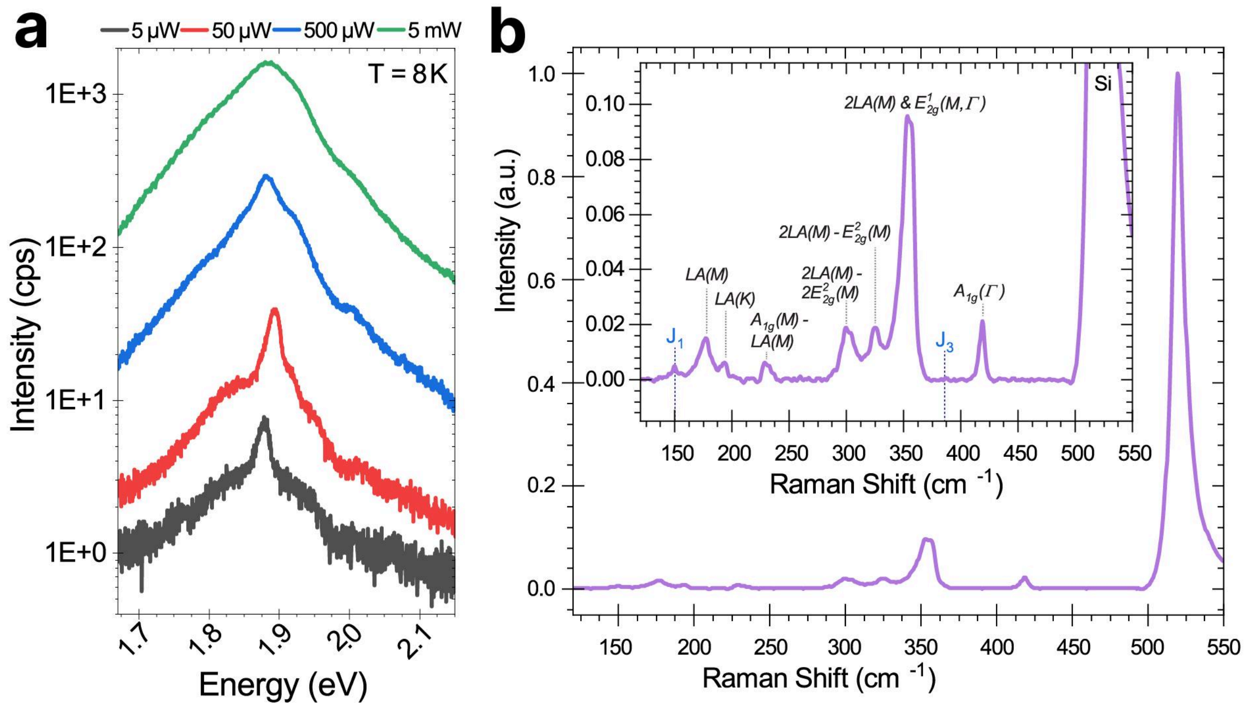

Supplementary Figure S18. Characterization of samples treated at 25 W for 10 s. (**a**) PL spectra at different powers shows a very low-intensity, yet non-saturating, signal at lower energy, but the *A* exciton and its complexes are not clearly resolvable. (**b**) Raman spectra of the 10s treated sample normalized to the silicon peak. In contrast to the Raman spectra in **Figure S5**, the WS_2 peaks are dwarfed by the silicon peak indicating that the sample quality is reduced. Inset shows zoomed in view of 1H and 1T peaks apparent in the spectra. While these results show that the phase mixture is still present in this longer treated sample, the quality of the flake has been degraded by the extended plasma treatment.

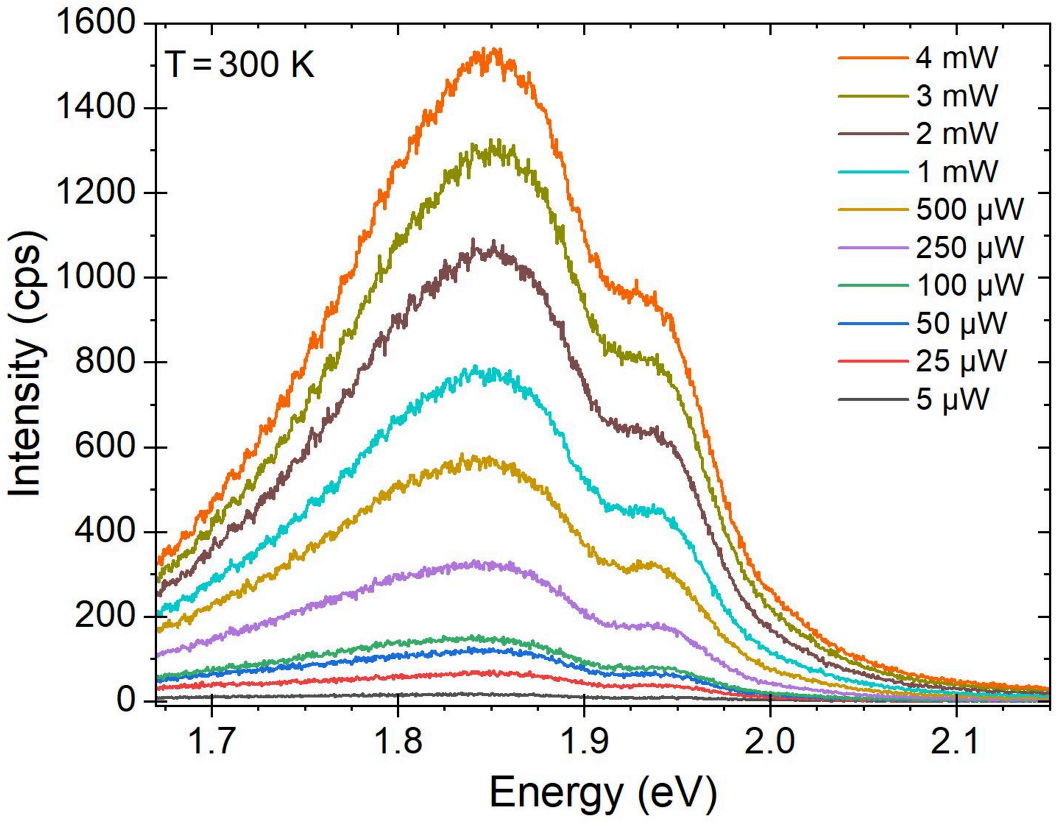

Supplementary Figure S19. Room temperature (T=300 K) power dependent PL spectra for 25 W 5 s treated WS_2 from **Figure 4** of the main text. Since the *M* band is observed at room temperature, its binding energy must be larger than thermal energy, $k_BT \approx 26$ meV.

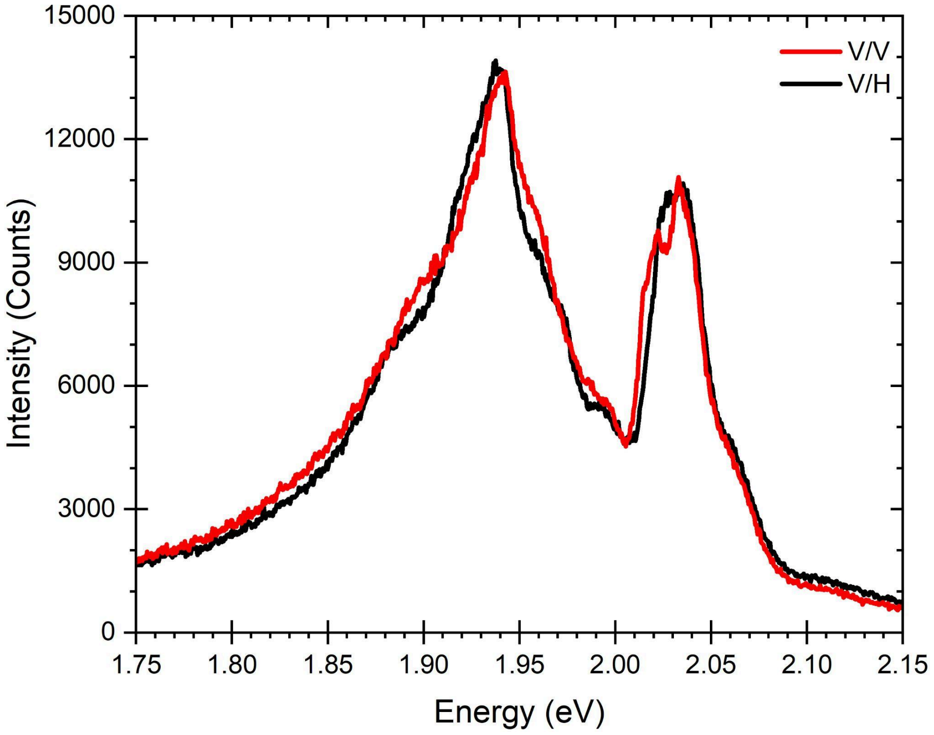

Supplementary Figure S20. Linear polarization of 25 W, 5 s treated sample. No significant difference is observed between the co- and cross-polarized spectra

Resonance	Degree of Spin Polarization
1T-L1: X_{M1}	95.589%
1T-L1: X_{M2}	94.113%
1T-Tr2 (Interface-I): X_M	95.691%
1T-Tr2 (Interface-II): X_M	99.998%

Supplementary Table S1. Degree of spin polarization of orbitals responsible for *M* band resonances in 1T-L1 and 1T-Tr2 supercells. For comparison, X_A & X_B in pristine 1H supercell have spin polarization > 99.9999999%.

References

(1) Yang, W.; Huang, T.; He, J.; Zhang, S.; Yang, Y.; Liu, W.; Ge, X.; Zhang, R.; Qiu, M.; Sang, Y.; Wang, X.; Zhou, X.; Li, T.; Liu, C.; Dai, N.; Chen, X.; Fan, Z.; Shen, G. Monolayer WS_2 Lateral Homosuperlattices with Two-Dimensional Periodic Localized Photoluminescence. *ACS Nano* **2022**, *16* (1), 597–603. https://doi.org/10.1021/acsnano.1c07803.

(2) Berkdemir, A.; Gutiérrez, H. R.; Botello-Méndez, A. R.; Perea-López, N.; Elías, A. L.; Chia, C.-I.; Wang, B.; Crespi, V. H.; López-Urías, F.; Charlier, J.-C.; Terrones, H.; Terrones, M. Identification of Individual and Few Layers of WS_2 Using Raman Spectroscopy. *Sci. Rep.* **2013**, *3* (1), 1755. https://doi.org/10.1038/srep01755.

(3) Han, A.; Zhou, X.; Wang, X.; Liu, S.; Xiong, Q.; Zhang, Q.; Gu, L.; Zhuang, Z.; Zhang, W.; Li, F.; Wang, D.; Li, L.-J.; Li, Y. One-Step Synthesis of Single-Site Vanadium Substitution in 1T-WS_2 Monolayers for Enhanced Hydrogen Evolution Catalysis. *Nat. Commun.* **2021**, *12* (1), 709. https://doi.org/10.1038/s41467-021-20951-9.

(4) Lin, Y.-C.; Yeh, C.-H.; Lin, H.-C.; Siao, M.-D.; Liu, Z.; Nakajima, H.; Okazaki, T.; Chou, M.-Y.; Suenaga, K.; Chiu, P.-W. Stable 1T Tungsten Disulfide Monolayer and Its Junctions: Growth and Atomic Structures. *ACS Nano* **2018**, *12* (12), 12080–12088. https://doi.org/10.1021/acsnano.8b04979.

(5) Ambrosi, A.; Sofer, Z.; Pumera, M. 2H → 1T Phase Transition and Hydrogen Evolution Activity of MoS_2 , $MoSe_2$, WS_2 and WSe_2 Strongly Depends on the MX_2 Composition. *Chem. Commun.* **2015**, *51* (40), 8450–8453. https://doi.org/10.1039/C5CC00803D.

(6) Wang, Z.; Liu, P.; Ito, Y.; Ning, S.; Tan, Y.; Fujita, T.; Hirata, A.; Chen, M. Chemical Vapor Deposition of Monolayer $Mo_{1-x}W_xS_2$ Crystals with Tunable Band Gaps. *Sci. Rep.* **2016**, *6* (1), 21536. https://doi.org/10.1038/srep21536.

(7) Plechinger, G.; Nagler, P.; Kraus, J.; Paradiso, N.; Strunk, C.; Schüller, C.; Korn, T. Identification of Excitons, Trions and Biexcitons in Single-Layer WS_2. *Phys. Status Solidi RRL – Rapid Res. Lett.* **2015**, *9* (8), 457–461. https://doi.org/10.1002/pssr.201510224.

(8) Paur, M.; Molina-Mendoza, A. J.; Bratschitsch, R.; Watanabe, K.; Taniguchi, T.; Mueller, T. Electroluminescence from Multi-Particle Exciton Complexes in Transition Metal Dichalcogenide Semiconductors. *Nat. Commun.* **2019**, *10* (1), 1709. https://doi.org/10.1038/s41467-019-09781-y.

(9) He, M.; Rivera, P.; Van Tuan, D.; Wilson, N. P.; Yang, M.; Taniguchi, T.; Watanabe, K.; Yan, J.; Mandrus, D. G.; Yu, H.; Dery, H.; Yao, W.; Xu, X. Valley Phonons and Exciton Complexes in a Monolayer Semiconductor. *Nat. Commun.* **2020**, *11* (1), 618. https://doi.org/10.1038/s41467-020-14472-0.

(10) Zinkiewicz, M.; Woźniak, T.; Kazimierczuk, T.; Kapuscinski, P.; Oreszczuk, K.; Grzeszczyk, M.; Bartoš, M.; Nogajewski, K.; Watanabe, K.; Taniguchi, T.; Faugeras, C.; Kossacki, P.; Potemski, M.; Babiński, A.; Molas, M. R. Excitonic Complexes in N-Doped WS_2 Monolayer. *Nano Lett.* **2021**, *21* (6), 2519–2525. https://doi.org/10.1021/acs.nanolett.0c05021.

(11) Chand, S. B.; Woods, J. M.; Quan, J.; Mejia, E.; Taniguchi, T.; Watanabe, K.; Alù, A.; Grosso, G. Interaction-Driven Transport of Dark Excitons in 2D Semiconductors with Phonon-Mediated Optical Readout. *Nat Commun* **2023**, *14*, 3712. https://doi.org/10.1038/s41467-023-39339-y.
